# Smoking and oral and pharyngeal cancer: a meta-analysis

**DOI:** 10.3389/or.2025.1672607

**Published:** 2026-01-20

**Authors:** Irene Possenti, Stefano Miotti, Silvano Gallus, Vincenzo Bagnardi, Werner Garavello, Claudia Specchia, Luc Smits, Anna Odone, Alessandra Lugo

**Affiliations:** 1 Department of Medical Epidemiology, Istituto di Ricerche Farmacologiche Mario Negri IRCCS, Milan, Italy; 2 Department of Statistics and Quantitative Methods, University of Milan-Bicocca, Milan, Italy; 3 Department of Otorhinolaryngology, Fondazione IRCCS San Gerardo Dei Tintori, Monza, Italy; 4 Department of Otorhinolaryngology, School of Medicine and Surgery, University of Milan-Bicocca, Monza, Italy; 5 Department of Molecular and Translational Medicine, University of Brescia, Brescia, Italy; 6 Department of Epidemiology, Care and Public Health Research Institute, Maastricht University, Maastricht, Netherlands; 7 School of Public Health, Department of Public Health, Experimental and Forensic Medicine, University of Pavia, Pavia, Italy; 8 Medical Direction, Fondazione IRCCS Policlinico San Matteo, Pavia, Italy

**Keywords:** oral cancer, pharyngeal cancer, oropharyngeal cancer, cigarette smoking, dose-response relationship, meta-analysis

## Abstract

**Introduction:**

Oral cavity and pharyngeal cancer (OPC) affects over 580,000 people globally each year, with tobacco and alcohol being key risk factors. This meta-analysis quantifies the excess risk of OPC associated with cigarette smoking and its patterns.

**Methods:**

We carried out a systematic review and meta-analysis of case-control and cohort studies assessing the association between cigarette smoking and OPC risk, including articles published up to February 2025. Using a combined umbrella and traditional review approach, we estimated pooled relative risks (RR) by smoking status, intensity, duration, and time since quitting.

**Results:**

Out of 137 eligible articles, 115 original studies were included in this meta-analysis. Relative to never smokers, the pooled risk of OPC was 3.58 (95% CI: 3.03–4.24; n = 54) among current smokers, 1.61 (95% CI: 1.44–1.81; n = 53) among former smokers, and 2.45 (95% CI: 2.19–2.75; n = 80) among ever smokers. Subsite-specific analyses showed RRs of 3.39 (95% CI: 2.64–4.35; n = 25) for oral cancer and 4.24 (95% CI: 2.96–6.09; n = 18) for pharyngeal cancer in current versus never smokers. Risk rose steeply with both smoking intensity and duration, doubling after 6 cigarettes per day or 7 years of smoking, before reaching a plateau around an RR of 5 at 20 cigarettes per day or 20 years. The risk declined linearly with longer time since quitting, with a 50% reduction observed within 10 years of cessation.

**Conclusion:**

Our findings reaffirm the substantial impact of smoking on OPC risk and stress the need for efforts to avoid smoking initiation and support cessation.

## Introduction

The annual worldwide incidence of oral cavity cancer is estimated at almost 400,000 cases, while pharyngeal cancer (excluding nasopharyngeal cancer) accounts for almost 200,000 new cases each year (1). In terms of mortality, an estimated 188,230 deaths occur annually due to oral cavity cancer, while pharyngeal cancer (excluding nasopharyngeal cancers) is associated with approximately 93,185 deaths per year (1). Nearly two-thirds of all new cases of oral cavity and pharyngeal cancer (OPC) each year arise in Asian countries, with the highest numbers reported in Sri Lanka, Indonesia, India, Pakistan and Bangladesh (2).

Several risk factors for the development of OPC have been identified in the scientific literature. Among these, the most studied are tobacco use and alcohol consumption (3, 4). Other important risk factors for OPC are the use of betel, a substance derived from the betel palm, which is widespread in South East Asia and increases the risk of OPC by more than 4 times (5), and human papillomavirus (HPV) infection. HPV infection has been estimated to increase the risk of OPC by more than 60% (6). In contrast, regular consumption of fruit and vegetables has been identified as a protective factor, reducing the risk of OPC by approximately 25% (7).

Trends in the disease in recent years have been influenced by changes in the lifestyles of different populations. In southern European men, where alcohol and tobacco consumption has declined since the mid-1980s, mortality from OPC has declined steadily over the years. In contrast, in Central and Eastern European countries, where consumption of these substances has remained high, deaths have increased in recent decades (8). In Asian countries, the number of OPC cases remains constant and very high (9), partly due to the large consumption of betel among the population (10).

Although the association between smoking and OPC is well established in literature, there is a lack of recent meta-analyses that focus on these cancer sites individually, rather than grouping them under the broader categories of head and neck cancer or upper aerodigestive tract cancer (11, 12).

The primary aim of this meta-analysis is to provide a precise quantification of the association between cigarette smoking and OPC risk, thereby addressing the gap left by recent meta-analyses on the topic. An additional goal is to compare subgroups based on sex, geographic area, and cancer anatomical subtype, amongst others, and to evaluate dose-response relationships, including smoking intensity, duration, and time since quitting.

## Methods

This work is part of a broader series of systematic reviews and meta-analyses investigating the link between cigarette smoking, second-hand smoke (SHS) exposure, and cancer risk (13–22). To ensure we identified all relevant original studies efficiently and thoroughly, we used a two steps approach (15). As a first step, we carried out an umbrella review to comprehensively identify meta-analyses, pooled analyses, and reviews that had examined the link between cigarette smoking and the risk of OPC. From these meta-analyses, pooled analyses and reviews, we extracted the original studies included in the identified reviews, allowing us to gather all high-quality evidence already available. Following this, a traditional review was undertaken to identify original studies published after the most recent comprehensive review. The study protocol is available in PROSPERO (registration number CRD42017063991).

The search strategy, inclusion criteria, and data extraction procedures are detailed in Annex 1.

We computed pooled relative risks (RR) for current, former, and ever smokers versus never smokers, both overall and separately by study type (cohort and case-control).

All analyses were performed using R software version 4.2.2 (R Development Core Team, 2017), utilizing the “meta” and “dosresmeta” packages for statistical computations.

## Results

### Study selection and description

Out of 137 eligible original articles, 22 were excluded as their participants overlapped with those reported in other included studies (Supplementary Table S2). Consequently, 115 original studies—comprising 87 case-control and 28 cohort designs—fulfilled the inclusion criteria and were incorporated into this meta-analysis (Supplementary Tables S3, S4). These studies, published between 1969 and 2024, encompassed data on over 40,000 cases of oral or pharyngeal cancer. Of these, 64 studies provided data specifically for oral cancer, 49 for pharyngeal cancer, and 36 studies provided only combined data for both anatomical subtypes.

A total of 54 studies on OPC reported risk estimates (or data allowing their calculation) for current smokers, 53 for former smokers, and 80 for ever smokers, all compared to never smokers. Additionally, 43 studies provided RR by smoking intensity (including 20 among current smokers), 30 by smoking duration (10 among current smokers), and 22 by time since quitting. Publications partially excluded from this meta-analysis, along with reasons for exclusion, are detailed in Supplementary Table S5. Quality assessments for case-control and cohort studies are presented in Supplementary Tables S6, S7, respectively, identifying 11 high-quality case-control and 14 high-quality cohort studies.

### Quantitative data synthesis

**FIGURE 1:**
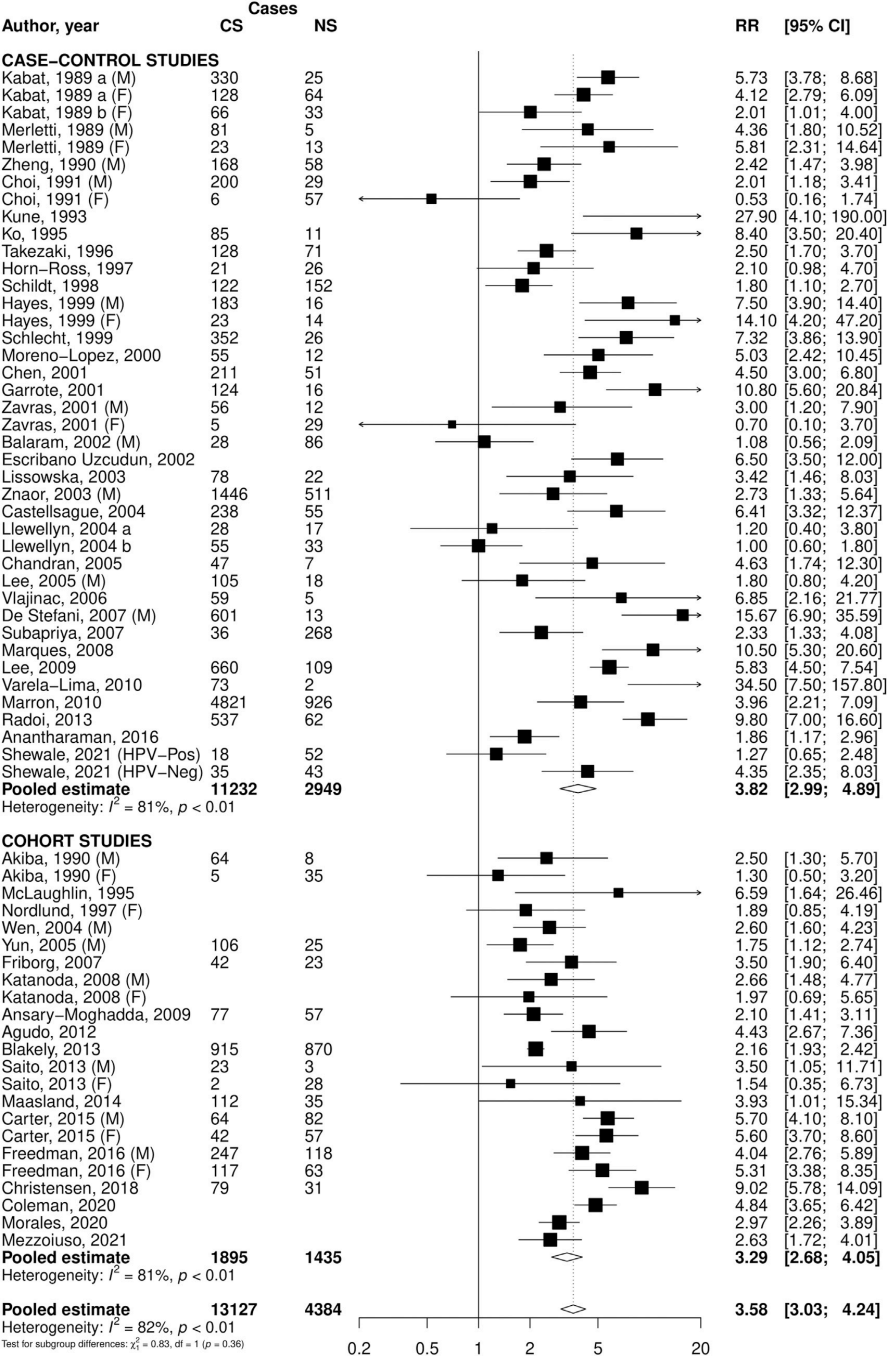
Forest plot of relative risks (RR) of oral and pharyngeal cancer for current cigarette smokers (CS) versus never smokers (NS), overall and stratified by study design. Footnote: CI: confidence interval; F: females; HPV+: Human Papillomavirus test positive subjects; HPV -: Human Papillomavirus test negative subjects; M: males.

**FIGURE 2:**
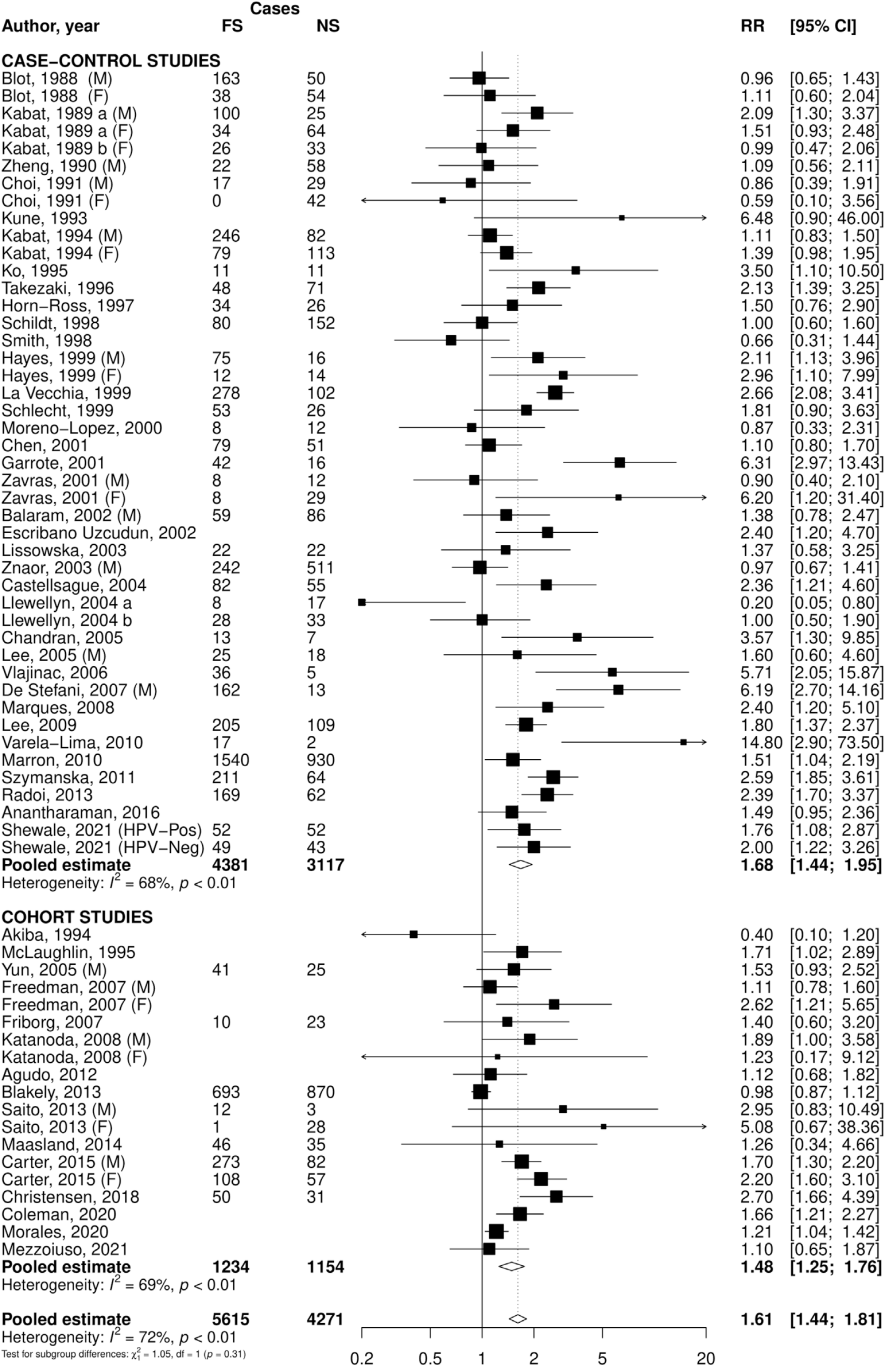
Forest plot of relative risks (RR) of oral and pharyngeal cancer for former cigarette smokers (FS) versus never smokers (NS), overall and stratified by study design. Footnote: CI: confidence interval; F: females; HPV+: Human Papillomavirus test positive subjects; HPV -: Human Papillomavirus test negative subjects; M: males.

The overall pooled RR of OPC for current smokers compared to never smokers was 3.58 (95% CI: 3.03–4.24), with case-control studies reporting a RR of 3.82 (95% CI: 2.99–4.89) and cohort studies 3.29 (95% CI: 2.68–4.05) (Figure 1). For former smokers versus never smokers, the pooled RR was 1.61 (95% CI: 1.44–1.81), with estimates of 1.68 (95% CI: 1.44–1.95) in case-control and 1.48 (95% CI: 1.25–1.76) in cohort studies (Figure 2). Among ever smokers, the overall RR was 2.45 (95% CI: 2.19–2.75), with 2.55 (95% CI: 2.21–2.94) in case-control and 2.15 (95% CI: 1.81–2.54) in cohort studies (Supplementary Figure S2). Significant heterogeneity was observed across all pooled estimates.

### Stratified analyses

The RRs stratified by anatomical subtype are reported in Supplementary Figures S3–S5 for oral cavity cancer (3.39 for current and 1.41 for former smokers) and in Supplementary Figures S6–S8 for pharyngeal cancer (4.24 for current and 1.94 for former smokers. Regarding pharyngeal subsites, the RR for oropharyngeal cancer was 3.20 for current smokers and 2.31 for former smokers. The RR for hypopharyngeal cancer was 4.21 for current smokers and 1.80 for former smokers.

Potential heterogeneity was further investigated through stratified analyses (Table 1). For current smokers, marked geographic differences emerged, with the highest RRs observed in South America (10.02) and Oceania (6.44), intermediate values in North America (5.01), Europe (3.59), and Africa (4.63, single study), and the lowest in Asia (2.26; p for subgroup differences <0.01). According to endpoint in cohort studies, significantly higher RR estimates were observed in studies analysing mortality than incidence, both for former and for ever smokers (RRs were 1.89 for mortality and 1.14 for incidence for former smokers, and 2.69 for mortality and 1.83 for incidence for ever smokers; p for subgroup differences <0.01).A significant difference was also observed among ever versus never smokers according to HPV serology (RRs were 3.28 for subjects with HPV negative serology and 1.35 for those with HPV positive serology; p-value for subgroup difference = 0.02). No significant variation emerged when stratifying by NOS quality score, sex, sample size, adequacy of confounder adjustment, geographic region, income level, or publication year.

**TABLE 1:** Pooled relative risk (RR) and corresponding 95% confidence interval (CI) for oral and pharyngeal (OPC) risk for current, former, and ever cigarette smokers vs. never cigarette smokers, overall and in strata of selected characteristics.

Strata	Current smokers				Former smokers				Ever smokers			
	N. studies	Pooled RR (95% CI)	p-value^a^	p-value^b^	N. studies	Pooled RR (95% CI)	p-value^a^	p-value^b^	N. studies	Pooled RR (95% CI)	p-value^a^	p-value^b^
Total	54	3.58 (3.03–4.24)	-	<0.01	53	1.61 (1.44–1.81)	-	<0.01	80	2.45 (2.19–2.75)	-	<0.01
Histological type												
Squamous cell	11	3.31 (2.07–5.30)	-	<0.01	9	1.86 (1.16–2.99)	-	<0.01	17	2.28 (1.71–3.03)	-	<0.01
Anatomical subtype												
Oral cavity	25	3.39 (2.64–4.35)	-	<0.01	23	1.41 (1.20–1.66)	-	<0.01	47	2.18 (1.89–2.52)	-	<0.01
Pharynx	18	4.24 (2.96–6.09)	-	<0.01	18	1.94 (1.41–2.67)	-	<0.01	30	3.25 (2.56–4.14)	-	<0.01
Oropharynx	5	3.20 (2.25–4.55)	-	0.07	4	2.31 (1.26–4.23)	-	0.12	12	2.94 (2.02–4.29)	-	<0.01
Hypopharynx	2	4.21 (0.78–22.59)	-	0.11	2	1.80 (0.20–16.00)	-	<0.01	8	3.95 (2.56–6.08)	-	<0.01
Type of study												
Case-control	36	3.82 (2.99–4.89)	0.36	<0.01	38	1.68 (1.44–1.95)	0.31	<0.01	62	2.55 (2.21–2.94)	0.13	<0.01
Cohort	18	3.29 (2.68–4.05)		<0.01	15	1.48 (1.25–1.76)		<0.01	18	2.15 (1.81–2.54)		<0.01
Sex												
Women	16	2.77 (1.89–4.07)	0.77	<0.01	14	1.50 (1.17–1.93)	0.38	0.06	17	2.05 (1.51–2.77)	0.36	<0.01
Men	22	2.98 (2.27–3.89)		<0.01	20	1.28 (1.00–1.65)		<0.01	29	2.47 (1.91–3.19)		<0.01
Type of controls^c^												
Hospital	21	3.93 (2.83–5.46)	0.76	<0.01	26	1.90 (1.55–2.32)	0.04	<0.01	33	2.47 (2.03–3.00)	0.58	<0.01
Population	11	3.59 (2.23–5.78)		<0.01	10	1.35 (1.04–1.75)		<0.01	22	2.72 (2.03–3.66)		<0.01
Endpoint^d^												
Incidence	9	3.00 (2.35–3.82)	0.45	<0.01	9	1.14 (0.99–1.31)	<0.01	0.12	13	1.83 (1.54–2.17)	<0.01	<0.01
Mortality	9	3.51 (2.53–4.86)		<0.01	6	1.89 (1.63–2.20)		0.66	6	2.69 (2.13–3.40)		0.08
HPV status												
Negative	-				-				4	3.28 (1.77–6.08)	0.02	<0.01
Positive									4	1.35 (0.90–2.02)		0.20
Number of cases^e^												
<126	20	3.11 (2.38–4.06)	0.43	<0.01	17	1.59 (1.06–2.38)	0.12	<0.01	27	2.34 (1.86–2.94)	0.16	<0.01
126–374	17	4.00 (2.89–5.54)		<0.01	16	1.89 (1.62–2.22)		0.04	29	2.29 (1.86–2.82)		<0.01
≥375	15	3.89 (2.82–5.11)		<0.01	19	1.49 (1.27–1.76)		<0.01	21	2.92 (2.42–3.51)		<0.01
Adjustements^f^												
Non-adequate	20	3.42 (2.67–4.38)	0.67	<0.01	17	1.49 (1.23–1.80)	0.32	<0.01	38	2.40 (2.01–2.86)	0.82	<0.01
Adequate	34	3.68 (2.93–4.62)		<0.01	36	1.68 (1.45–1.94)		<0.01	42	2.46 (2.13–2.84)		<0.01
Adjustement for betel quid or tobacco chewing^g^												
Non-adjusted	9	2.26 (1.91–2.68)	0.81	0.42	8	1.47 (1.11–1.94)	0.11	0.18	20	2.20 (1.76–2.77)	0.44	<0.01
Adjusted	5	2.45 (1.31–4.59)		<0.01	4	1.36 (0.87–2.12)		0.15	14	2.53 (1.95–3.28)		0.003
Study quality												
Low (NOS<7)	43	3.64 (2.94–4.50)	0.91	<0.01	41	1.67 (1.45–1.92)	0.31	<0.01	62	2.48 (2.16–2.85)	0.58	<0.01
High (NOS≥7)	11	3.70 (3.00–4.58)		<0.01	12	1.47 (1.20–1.79)		<0.01	16	2.32 (1.94–2.78)		0.05
Geographic area^h^												
North America	11	5.01 (3.96–6.35)	<0.01	<0.01	14	1.60 (1.35–1.90)	0.28	<0.01	15	2.47 (2.06–2.96)	0.25	<0.01
Europe	19	3.59 (2.63–4.89)		<0.01	18	1.59 (1.24–2.04)		<0.01	18	2.58 (1.88–3.55)		<0.01
Asia	14	2.26 (1.91–2.67)		0.08	12	1.41 (1.12–1.78)		0.11	34	2.33 (1.97–2.77)		<0.01
South America	3	10.02 (6.68–15.03)		0.35	3	2.91 (1.44–5.88)		0.07	4	5.12 (2.85–9.19)		0.17
Oceania	2	6.44 (0.54–76.97)		<0.01	2	1.93 (0.33–11.42)		0.06	2	3.87 (0.38–39.30)		0.02
Africa	1	4.63 (1.74–12.31)		-	1	3.57 (1.30–9.83)		-	3	2.19 (0.88–5.45)		0.10
Income group^i^												
High income	42	3.60 (3.01–4.31)	0.50	<0.01	43	1.54 (1.37–1.73)	0.07	<0.01	52	2.46 (2.16–2.79)	0.83	<0.01
Middle or low income	8	4.47 (2.48–8.05)		<0.01	8	2.38 (1.51–3.77)		<0.01	26	2.38 (1.86–3.05)		<0.01
Year of publication												
<2000	15	3.41 (2.49–4.66)	0.57	<0.01	17	1.44 (1.19–1.74)	0.35	<0.01	24	2.46 (1.91–3.17)	0.99	<0.01
2000–2009	23	3.35 (2.50–4.49)		<0.01	21	1.74 (1.35–2.25)		<0.01	23	2.48 (1.96–3.13)		<0.01
≥2010	15	4.06 (3.11–5.23)		<0.01	15	1.70 (1.42–2.04)		<0.01	33	2.43 (2.09–2.83)		<0.01

^a^ p-value for heterogeneity across strata.

^b^ p-value for heterogeneity within strata.

^c^ Type of controls for case-control studies only. Studies considering both studies with hospital and with population controls were not included in this stratification.

^d^ Endpoint for cohort studies only. Studies providing RRs, for both incidence and mortality, separately, were considered in both categories.

^e^ Studies in which the number of cases was not reported were not included in this stratification.

^f^ Estimates adjusted for, at least, age, sex, and alcohol consumption.

^g^ Restricted to studies conducted in Asia.

^h^ Studies conducted in multiple countries from different geographic areas were not included in this stratification.

^i^ Studies conducted in multiple countries with different income groups were not included in this stratification.

NOS: Newcastle Ottawa Scale.

### Publication bias

No indication of publication bias was observed for current smoking and OPC risk, based on both funnel plot inspection (Supplementary Figure S9; panel A) and Egger’s test (p = 0.06). In contrast, evidence of bias emerged for former and ever smokers (p = 0.01 and p = 0.03; panels B and C, respectively). Adjusted pooled estimates obtained using the Duval and Tweedie trim-and-fill method are reported in Supplementary Table S8.

### Dose-response analysis

**FIGURE 3:**
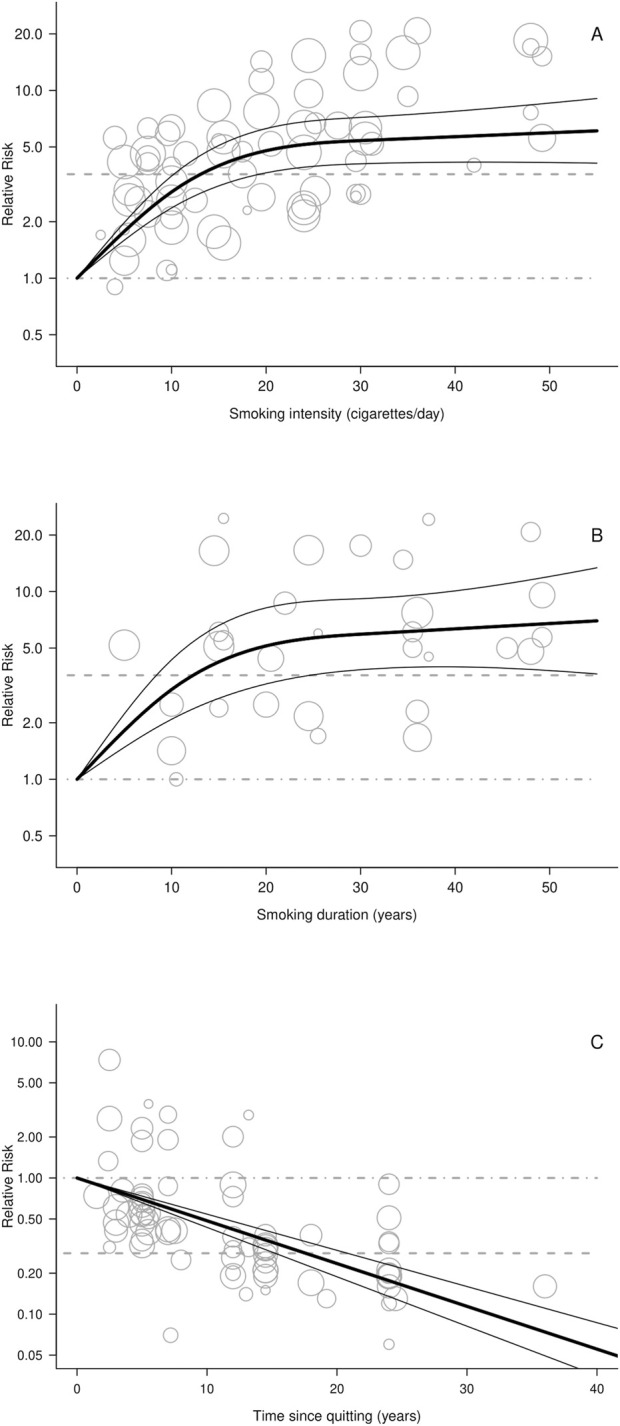
Relative risk (RR) curves illustrating the dose-response relationships between cigarette smoking and oral and pharyngeal cancer risk: **(A)** smoking intensity (current vs never smokers); **(B)** smoking duration (current vs never smokers); and **(C)** time since quitting smoking (former vs current smokers). **Footnote:** A: cigarette smoking intensity (based on 20 studies); B: cigarette smoking duration (based on 10 studies); C: time since quitting (based on 21 studies). 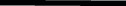
 Restricted cubic spline from random-effects dose-response models **(A,B)** or linear model **(C)**; 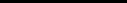
 95% confidence interval of the spline models; 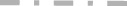
 RR for the reference category (never smokers in **(A**,**B)**, current smokers in **(C)**); 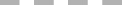
 RR for current vs. never cigarette smokers **(A**,**B)** former vs. current cigarette smokers **(C)**; 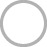
 RR for various exposure categories in each study included in the analysis. The area of the circle is proportional to the precision (i.e. to the inverse variance) of the RR.

Figure 3 illustrates the dose–response relationships between smoking intensity (panel A), duration (panel B), and time since quitting (panel C) and OPC risk. Among current smokers, OPC risk rose nonlinearly with intensity, increasing sharply between 5 and 10 and 20 cigarettes/day before plateauing. Estimated RRs were 1.98 (95% CI: 1.74–2.25) for six, 2.88 (95% CI: 2.37–3.50) for ten, and 4.78 (95% CI: 3.64–6.27) for 20 cigarettes/day (estimated using the curve functions reported in Supplementary Box 2). A nonlinear trend was also observed for smoking duration, with RRs of 2.01 (95% CI: 1.62–2.51) after seven, 2.64 (95% CI: 1.96–3.57) after ten, and 5.10 (95% CI: 3.27–7.96) after 20 years. Conversely, OPC risk declined with time since quitting, with the RR halving within 10 years (0.51; 95% CI: 0.42–0.64) and approaching that of never smokers after 18 years (0.21; 95% CI: 0.17–0.33).

## Discussion

This systematic review and meta-analysis offers the most up-to-date and comprehensive evidence on the relationship between cigarette smoking and oral and pharyngeal cancer (OPC). Drawing on 137 eligible publications and 115 original studies covering more than 40,000 OPC cases, our results indicate that current smokers have over a threefold higher risk of OPC compared with never smokers, while former smokers retain a 60% increased risk. Dose-response analyses demonstrated that OPC risk more than doubles with as few as six cigarettes per day or after only 7 years of smoking. The risk declines substantially after quitting, with former smokers reaching half the risk within 7 years of cessation.

From a public health perspective, the strong association between smoking and OPC risk underscores the need for reinforced tobacco control measures. In particular, tailored smoking cessation programs and awareness campaigns should be prioritized, especially in regions with the highest disease burden. Increasing tobacco prices through taxation represents one of the most effective strategies to reduce smoking prevalence and related health risks, particularly among young people and low-income populations (23). These interventions are essential to prevent OPC and other smoking-related cancers. The most recent and comprehensive meta-analysis focusing on the association between smoking and the risk of OPC, which was published in 2009, reported an increased risk (RR 3.41) similar to that found in our study (24). That meta-analysis included 25 original articles; therefore our study, which is based on about 50% more articles, strengthens the robustness of these findings.

By relating our RR estimates for current vs. never and former vs. never smokers, we obtain a former vs. current RR of 0.45. This estimate aligns closely with the RR of 0.44 reported in a recent meta-analysis on smoking cessation (25), further reinforcing the consistency of our findings and strengthening benefits of quitting smoking.

Our stratified analysis by anatomical subtype showed differences in the risk of OPC associated with cigarette smoking. For current smokers compared with never smokers, the pooled RR of oral cancer was 3.39, which is similar to the 3.43 RR reported in the 2008 meta-analysis conducted by Gandini et al. (4). Although more recent meta-analyses have examined the association between smoking and oral cancer, they have focused on specific populations or histological subtypes, limiting the generalisability of their findings (26, 27). On the other hand, the association between smoking and pharyngeal cancer has been less extensively investigated in previous meta-analyses, with the only one reporting an RR for pharyngeal cancer being Gandini’s 2008 study (4). Notably, the RR reported in this meta-analysis (RR 6.76) was significantly higher than our estimate (RR 4.24), but it was based on only seven studies, while we found 43 studies investigating pharyngeal cancer. It has been hypothesized that the higher risk associated with smoking for pharyngeal cancer compared to oral cancer is due to the aerodynamics of respiratory flow in the upper airway, as hypothesized in a previous study (28). By providing an updated and comprehensive quantification of risk estimates for both cancer subtypes, this meta-analysis fills a critical gap in the literature.

Further stratification of pharyngeal cancer into oropharyngeal and hypopharyngeal cancer showed pooled RRs of 2.94 and 3.63, respectively, for ever versus never smokers. A previous meta-analysis investigating this difference reported a higher RR for oropharyngeal than hypopharyngeal cancer (29). However, its estimates were based on a much smaller number of studies (3 for oropharynx and 2 for hypopharynx) compared to our analysis (12 and 8, respectively). The previously hypothesised association between airflow dynamics and higher smoking-related risk in the oropharynx compared with the oral cavity (28) may also explain the difference between subtypes, as the oropharynx, being closer to the oral cavity, may reflect a decreasing exposure gradient along the airway. Notwithstanding this, evidence remains scarce, with estimates for current and former smokers based on only two studies. The limited data on these subtypes highlights a critical gap in the literature and affects the generalizability of our results, underscoring the need for additional well-designed studies focusing specifically on oropharyngeal and hypopharyngeal cancers.

To our knowledge, this is the first meta-analysis to provide risk estimates for oral and pharyngeal squamous cell carcinoma associated with smoking. The risks we found for current, former, and ever smokers for the oral and pharyngeal squamous cell carcinoma appear to be quite similar to the overall estimates for OPC. This observation contrasts with findings for other cancer sites, such as nasopharynx and lung, where higher risks associated with smoking have been documented for squamous cell carcinoma (18, 30, 31). The lack of such difference in our results may indicate a more uniform carcinogenic effect of smoking throughout the oral cavity and pharynx, suggesting the need for further investigation into the underlying biological mechanisms and potential risk factors contributing to this phenomenon.

Our analysis shows significant differences in the risks of OPC for different endpoints in cohort studies, with higher RR estimates for mortality compared to incidence. The increased mortality risk associated with smoking suggests that not only the incidence, but also the prognosis of individuals diagnosed with these cancers is worse in smokers than in never smokers, highlighting the urgent need for effective cessation strategies and early detection in this high-risk population.

When we stratified by HPV serological status, we found that the risk associated with smoking was substantially higher among HPV-negative individuals than among HPV-positive individuals. HPV infection is one of the main etiological factors for cancers of the oral cavity and pharynx, particularly tumours arising in the oropharynx (6). Consequently, in HPV-positive individuals, tobacco smoking’s relative contribution to cancer risk may appear diluted, as HPV-driven oncogenic pathways tend to dominate tumour development (32). Importantly, just few studies provided the stratification by HPV serological status. Future studies should therefore consider HPV status in their analyses and stratify or adjust for it appropriately, as failure to do so may mask important differences in risk magnitude across subgroups.

Significant geographic variations in risk estimates were observed for current smokers, with the lowest risks reported in studies from Asia. Non-significantly lower RRs were found in Asia compared with other regions also among former and ever smokers. These findings are in line with a previous meta-analysis focused exclusively on oral cancer, which reported a RR of 1.88 in Asia, compared with 3.62 in Africa, 7.65 in the Americas, and 3.12 in Europe (33). Given that around two-thirds of all OPC cases occur in Asia (2), it is important to investigate other risk factors for OPC that are particularly prevalent in this region, such as betel quid and areca-nut consumption, which plays major roles in OPC etiology and may confound the association with smoking (5). For instance, use of areca nut has been linked with oral cancer risks of approximately 7.9 in South-Central Asia (34). However, in our stratified analysis restricted to Asian studies, no significant differences emerged in the pooled risk estimates between studies that adjusted for betel quid or tobacco chewing and those that did not, suggesting that these factors may not fully explain the lower RRs observed in Asian populations. Other regional factors, such as differences in smoking habits and cultural patterns on tobacco use, including the frequent use of bidis (35), could result in lower exposure to mainstream smoke compared with manufactured cigarettes and partially explain the observed geographic difference. Genetic or epigenetic factors specific to Asian populations may also modulate susceptibility to the carcinogenic effects of tobacco. In fact, some studies have identified gene–environment interactions and biomarker profiles unique to Asian populations that may influence OPC risk (36, 37). In contrast, the exceptionally high RRs reported in Africa, Oceania, and South America are likely influenced by the limited number of available studies, which may distort overall risk estimates. This underlines the clear need for further research in these areas in order to better characterise the epidemiology of smoking-related cancers and to inform more accurate and region-specific public health strategies. Moreover, when using relative risks to estimate population attributable fractions (PAF) for OPC, region-specific RRs should be applied, as pooling global estimates may dilute the PAFs in Western regions where smoking-related risks are substantially higher.

The dose-response analysis revealed a nonlinear association for smoking intensity, with a steep increase in risk up to 20 cigarettes per day, followed by a plateau. This highlights the fact that even smoking a small number of cigarettes per day, e.g., six, is sufficient to double the risk of OPC. A similar nonlinear pattern was observed for smoking duration, with the risk doubling after as little as 7 years. time since quitting showed an inverse linear relationship with OPC risk: the risk was halved within 7 years of cessation and approached that of never smokers after 18 years. This finding, consistent with results from a recent meta-analysis (25), carries strong public health implications, underscoring the importance of quitting as early as possible. A significant and relevant decrease in OPC risk is observed even for few years since smoking cessation, thus suggesting that quitting smoking is always beneficial.

Our systematic review and meta-analysis present several notable strengths. By combining an umbrella review with a traditional review (15), we applied an efficient and effective methodology that enabled the inclusion of over 100 epidemiological studies on cigarette smoking and OPC risk, making this the most comprehensive meta-analysis to date on the topic. Careful screening of all retrieved publications was conducted to prevent data overlap. Study quality was thoroughly assessed using the NOS (38). Although no study was excluded in order to preserve comprehensiveness, stratified analyses by NOS score showed no significant differences in OPC risk estimates for current, former, and ever smokers. Furthermore, we applied flexible random-effects models with restricted cubic splines (39) to capture the dose–response relationships for smoking intensity, duration, and time since quitting, providing a more precise characterization of risk patterns.

This study shares the common limitations of meta-analyses based on epidemiological research. Both case-control and cohort designs are susceptible to selection and recall biases. Since smoking status, intensity, duration, and cessation time were self-reported across all studies, some degree of differential misclassification of exposure may have occurred. We believe the impact of these biases to be limited, as risk estimates did not vary significantly by study design. While it has been suggested that hospital-based controls may over-represent smokers - potentially biasing results toward the null (40) - our findings do not support this, given the absence of significant differences by control type in case-control studies. However, our results do not support this hypothesis, as no significant differences according to type of controls emerged among case-control studies. Substantial heterogeneity was observed across all smoking status analyses, likely reflecting variation in study methodologies and participant characteristics. Although random-effects models were applied to address this heterogeneity, it was not fully eliminated. Stratified analyses considering cancer anatomical subtypes, socioeconomic factors, and study features were conducted to explore potential sources of heterogeneity, but these factors only partly accounted for the observed variability. Finally, evidence of publication bias was detected in the analyses on former and ever smokers. To account for this, we applied the trim-and-fill method (41), which slightly lowered the pooled relative risk estimates, suggesting that selective publication of positive findings may have partially inflated the observed associations.

In conclusion, this meta-analysis reaffirms the strong association between cigarette smoking and the risk of OPC, demonstrating that even a moderate smoking intensity and a short smoking duration can substantially elevate this risk. These findings emphasise the urgent need for immediate, coordinated action from policymakers, healthcare professionals, and researchers to curb smoking-related cancer risks. Strengthening prevention policies, ensuring sustained investment in tobacco control, and promoting cessation at both individual and population levels are essential steps to reduce the future burden of OPC.

## Data Availability

The original contributions presented in the study are included in the article/Supplementary Material, further inquiries can be directed to the corresponding author.
