# Supplementary material for: Smoking and oral and pharyngeal cancer: a meta-analysis
Source: Oncol Rev. 2026 Jan 20;19:1672607. doi: 10.3389/or.2025.1672607 (PMC12865294; doi:10.3389/or.2025.1672607)
Supplement: Supplementary file 1 [file Supplementaryfile1.docx]

**SUPPLEMENTARY MATERIAL**

| **Description of tables or figures** | **Page** |
| --- | --- |
| **Supplementary Table 1.** List of 61 included meta-analysis, pooled-analyses, systematic reviews, and reports on the association between smoking and upper aerodigestive cancer risk. | 3 |
| **Supplementary Table 2.** List of 116 excluded publications (94 ineligible and 22 with duplicated results) for the review and meta-analysis and reason for exclusion. | 5 |
| **Supplementary Table 3.** Main characteristics of the 87 case-control studies on the association between cigarette smoking and oral and pharyngeal cancer risk included in the review, and corresponding information contributing to the meta-analysis. | 9 |
| **Supplementary Table 4.** Main characteristics of the 28 cohort studies on the association between cigarette smoking and oral and pharyngeal cancer risk included in the review and corresponding information contributing to the meta-analysis. | 21 |
| **Supplementary Table 5.** List of publications containing data that was partially excluded from the meta-analysis and reason for exclusion. | 24 |
| **Supplementary Table 6.** Quality evaluation of the 87 case-control studies included in the present meta-analysis using the New-Castle Ottawa (NOS) scale. | 27 |
| **Supplementary Table 7.** Quality evaluation of the 28 cohort studies included in the present meta-analysis using the New-Castle Ottawa (NOS) scale. | 32 |
| **Supplementary Figure 1.** Flowchart for the selection of the original studies on the association between cigarette smoking and oral and pharyngeal cancer risk included in the review and meta-analysis. | 35 |
| **Supplementary Figure 2**. Forest plot of study-specific and pooled relative risk (RR) of oral and pharyngeal cancer for ever smokers (ES) versus never smokers (NS), overall and by study design. | 36 |
| **Supplementary Figure 3**. Forest plot of study-specific and pooled relative risk (RR) of oral cancer for current smokers (CS) versus never smokers (NS), overall and by study design. | 37 |
| **Supplementary Figure 4**. Forest plot of study-specific and pooled relative risk (RR) of oral cancer for former smokers (FS) versus never smokers (NS), overall and by study design. | 38 |
| **Supplementary Figure 5**. Forest plot of study-specific and pooled relative risk (RR) of oral cancer for ever smokers (ES) versus never smokers (NS), overall and by study design. | 39 |
| **Supplementary Figure 6**. Forest plot of study-specific and pooled relative risk (RR) of pharyngeal cancer for current smokers (CS) versus never smokers (NS), overall and by study design. | 40 |
| **Supplementary Figure 7**. Forest plot of study-specific and pooled relative risk (RR) of pharyngeal cancer for former smokers (FS) versus never smokers (NS), overall and by study design. | 41 |
| **Supplementary Figure 8**. Forest plot of study-specific and pooled relative risk (RR) of pharyngeal cancer for ever smokers (ES) versus never smokers (NS), overall and by study design. | 42 |
| **Supplementary Figure 9.** Funnel plot of studies on the association between current (panel A), former (panel B), and ever (panel C) cigarette smokers versus never smokers and oral and pharyngeal cancer risk. | 43 |
| **Supplementary Table 8.** Comparison of original and trim-and-fill adjusted pooled relative risk (RR) and corresponding 95% confidence interval (CI) for oral and pharyngeal (OPC) risk for current, former, and ever cigarette smokers vs. never cigarette smokers. | 44 |
| **Supplementary Box 1.** Literature search strings for the update of the last available comprehensive review on the association between smoking and oral and pharyngeal cancer risk used in MEDLINE and Embase. | 45 |
| **Supplementary Box 2.** Functions of the splines and linear models used to estimate the associations between smoking intensity (current vs. never smokers), duration (current vs. never smokers) and time since quitting (former vs. current smokers) and the risk of oral and pharyngeal cancer. | 46 |
| **References** | 47 |

**Supplementary Table 1.** List of 61 included meta-analysis, pooled-analyses, systematic reviews, and reports on the association between smoking and upper aerodigestive cancer risk.

| **First Autor, Year** | **Type of analysis** |
| --- | --- |
| Ansary-Moghaddam, 2009 [1] | Meta-analysis |
| Ansary-Moghaddam, 2009 [2] | Pooled-analysis |
| Asombang, 2019 [3] | Review |
| Bakkalci, 2020 [4] | Review |
| Berthiller, 2016 [5] | Pooled-analysis |
| Carter, 2015 [6] | Pooled-analysis |
| Castellsagué, 1999 [7] | Pooled-analysis |
| Castellsagué, 2000 [8] | Pooled-analysis |
| Castro, 2018 [9] | Meta-analysis |
| Chang, 2019 [10] | Pooled-analysis |
| Chetwood, 2019 [11] | Review |
| Cook, 2010 [12] | Pooled-analysis |
| Di Credico, 2019 [13] | Pooled-analysis |
| Drahos, 2016 [14] | Pooled-analysis |
| Du, 2018 [15] | Review |
| Fahey, 2015 [16] | Meta-analysis |
| Gandini, 2008 [17] | Meta-analysis |
| Hashibe, 2007 [18] | Pooled-analysis |
| Hashibe, 2009 [19] | Pooled-analysis |
| IARC, 2004 [20] | Report |
| IARC, 2012 [21] | Report |
| Ishikawa, 2006 [22] | Pooled-analysis |
| Jia, 2012 [23] | Review |
| Jones, 2013 [24] | Meta-analysis |
| Khalifeh, 2024 [25] | Meta-analysis |
| Katanoda, 2008 [26] | Pooled-analysis |
| Khani, 2018 [27] | Review |
| Koyanagi, 2016 [28] | Meta-analysis |
| Lin, 2021 [29] | Pooled-analysis |
| Long, 2017 [30] | Meta-analysis |
| Lubin, 2009 [31] | Pooled-analysis |
| Lubin, 2012 [32] | Pooled-analysis |
| Macfarlane, 1995 [33] | Pooled-analysis |
| Mello, 2019 [34] | Meta-analysis |
| Miyazaki, 2017 [35] | Meta-analysis |
| Nakamura, 2009 [36] | Meta-analysis |
| Okekpa, 2019 [37] | Meta-analysis |
| Ordóñez-Mena, 2016 [38] | Pooled-analysis |
| Oze, 2012 [39] | Meta-analysis |
| Oze, 2019 [40] | Pooled-analysis |
| Romdhoni, 2023 [41] | Meta-analysis |
| Park, 2014 [42] | Meta-analysis |
| Petti, 2013 [43] | Meta-analysis |
| Prabhu, 2013 [44] | Meta-analysis |
| Prabhu, 2014 [45] | Meta-analysis |
| Prasad, 2019 [46] | Meta-analysis |
| Sadri, 2007 [47] | Meta-analysis |
| Saito, 2017 [48] | Pooled-analysis |
| t Mannetje, 1999 [49] | Pooled-analysis |
| Toporcov, 2015 [50] | Pooled-analysis |
| Tramacere, 2011 [51] | Meta-analysis |
| SGR, 2001 [52] | Report |
| SGR, 2004 [53] | Report |
| Wang, 2017 [54] | Meta-analysis |
| Wyss, 2013 [55] | Pooled-analysis |
| Xue, 2013 [56] | Meta-analysis |
| Yu, 2014 [57] | Meta-analysis |
| Zeka, 2003 [58] | Meta-analysis |
| Zhang, 2011 [59] | Meta-analysis |
| Zheng, 2014 [60] | Pooled-analysis |
| Zuo, 2017 [61] | Meta-analysis |

IARC: International Agency for Research on Cancer; SGR: Surgeon General Report.

**Supplementary Table 2.** List of 115 excluded publications (94 ineligible and 22 with duplicated results) for the review and meta-analysis and reason for exclusion.

| **First Author, Year** | **Study design** | **Reason** |
| --- | --- | --- |
| **Ineligible articles** | | |
| Allen, 2009 [62] | CO | No RR |
| Anantharaman, 2007 [63] | CC | Specific populations |
| Andrade, 2015 [64] | CC | Not in english |
| Andreotti, 2006 [65] | CC | Not in english |
| Benhamou, 2004 [66] | CC | Specific populations |
| Boffetta, 1992 [67] | CC | Wrong reference category |
| Bosetti, 2003 [68] | CC | Wrong reference category |
| Bravi, 2013 [69] | CC | Wrong reference category |
| Bross, 1976 [70] | CC | Wrong reference category |
| Brugere, 1986 [71] | case-only | Controls with cancer |
| Buch, 2002 [72] | CC | Specific populations |
| Canova, 2010 [73] | CC | No RR |
| Cha, 2007 [74] | CC | No RR |
| Chang, 2011 [75] | CC | Wrong reference category |
| Chang, 2019 [10] | PA | Wrong reference category |
| Chatterjee, 2010 [76] | CC | Specific populations |
| Choi, 1992 [77] | NA | Not in english |
| Chyou, 1995 [78] | CO | No RR |
| Cordero, 2010 [79] | CC | Wrong reference category |
| Cui, 2006 [80] | CC | No RR |
| De Stefani, 1992 [81] | CC | Wrong reference category |
| Di Credico, 2019 [13] | PA | No RR |
| Divaris, 2010 [82] | CC | No RR |
| Doll, 2005 [83] | CO | No RR |
| Elwood, 1984 [84] | CC | Controls with cancer |
| Galli, 2009 [85] | CC | No RR |
| Gronau, 2003 [86] | CC | No RR |
| Guha, 2007 [87] | CC | No RR |
| Guo, 2012 [88] | CC | Not in english |
| Hammond, 1958 [89] | CO | No RR |
| Hashibe, 2006 [90] | CC | No RR |
| Hashibe, 2007 [91] | PA | Specific populations |
| Henderson, 1976 [92] | CC | No RR |
| Hirayama, 1990 [93] | NA | Book or symposium |
| Idris, 1991 [94] | CO | Other tobacco products |
| Jaber, 1999 [95] | CC | Non inherent |
| Jayaprakash, 2006 [96] | CC | No RR |
| Kabat, 1994 [97] | CC | Other tobacco products |
| Kamiyama , 2005 [98] | NA | Not in english |
| Kao, 2002 [99] | CC | No RR |
| Keller, 1965 [100] | CC | No RR |
| La Vecchia, 1991 [101] | CC | No RR |
| La Vecchia, 1999 [102] | CC | Letter |
| Lee, 2009 [103] | CO | Specific populations |
| Lee, 2017 [104] | CO | Specific populations |
| Levi, 1998 [105] | CC | No RR |
| Lin, 2011 [106] | CC | Wrong reference category |
| Lohe, 2010 [107] | CC | No RR |
| Losi-Guembarovski, 2008 [108] | CC | No RR |
| Lubin, 2009 [31] | PA | No RR |
| Macfarlane, 1995 [33] | PA | No RR |
| Maier, 1992 [109] | CC | Wrong reference category |
| Maier, 1994 [110] | CC | Wrong reference category |
| Mashberg, 1993 [111] | CC | Controls with cancer |
| Menvielle, 2004 [112] | CC | No RR |
| Moura, 2014 [113] | CC | Controls with cancer |
| Nasher, 2014 [114] | CC | No RR |
| Notani, 1988 [115] | CC | Other tobacco products |
| Oreggia, 1991 [116] | CC | No RR |
| Pacella-Norman, 2002 [117] | CC | Controls with cancer |
| Peters, 2006 [118] | CC | Wrong reference category |
| Preston-Martin, 1988 [119] | CC | No RR |
| Randi, 2007 [120] | CC | Other tobacco products |
| Rao, 1994 [121] | CC | Wrong reference category |
| Ray, 2013 [122] | CC | No RR |
| Rogers, 1993 [123] | CC | No RR |
| Rosenblatt, 2004 [124] | CC | Wrong reference category |
| Rosenquist, 2005 [125] | CC | Not in english |
| Sanderson, 1997 [126] | CC | Wrong reference category |
| Sankaranarayanan, 1989 [127] | CC | Wrong reference category |
| Sankaranarayanan, 1990 [128] | CC | Controls with cancer |
| Sato, 1999 [129] | CC | No RR |
| Sato, 2011 [130] | CC | No RR |
| Sharma, 2006 [131] | CC | No RR |
| Shiu, 2004 [132] | CC | No RR |
| Shukla, 2012 [133] | CC | No RR |
| Singh, 2014 [134] | CC | No RR |
| Smith, 2004 [135] | CO | Specific populations |
| Sreelekha, 2001 [136] | CC | No RR |
| Suzuki, 2006 [137] | CC | No RR |
| Talamini, 1990 [138] | CC | Specific populations |
| Talamini, 1998 [139] | CC | Specific populations |
| Thomas, 2007 [140] | CC | Other tobacco products |
| Tsai, 2009 [141] | CC | No RR |
| Vincent, 1963 [142] | CC | No RR |
| Weikert, 2009 [143] | CO | No RR |
| Weir, 1970 [144] | CO | No RR |
| Williams, 1977 [145] | CO | Controls with cancer |
| Wynder, 1977 [146] | CC | Wrong reference category |
| Yang, 2007 [147] | CO | Wrong reference category |
| Yen, 2008 [148] | CO | Wrong reference category |
| Zhang, 2005 [149] | CC | No RR |
| Zheng, 1990 [150] | CC | No RR |
| Zheng, 1992 [151] | CC | No RR |
| **Eligible articles** | | |
| Anantharaman, 2011 [152] | CC | Included in Lee, 2009 [153] |
| Baron, 1993 [154] | CC | Included in La Vecchia, 1999 [155] and [19] |
| Barra, 1991 [156] | CC | Included in Bosetti, 2008 [157] |
| Bosetti, 2000 [158] | CC | Included in La Vecchia, 1999 [155] and [5] |
| Bosetti, 2008 [157] | CC | Included in La Vecchia, 1999 [155] and [5] |
| De Stefani, 1998 [159] | CC | Included in De Stefani, 2007 [160] |
| Ferraroni, 1989 [161] | CC | Included in Bosetti, 2008 [157] |
| Franceschi, 1992 [162] | CC | Included in Bosetti, 2008 [157] |
| Franceschi, 1999 [163] | CC | Included in La Vecchia, 1999 [155] and [164] |
| Galeone, 2015 [165] | PA | Included in Toporcov, 2015 [50] |
| Herrero, 2003 [166] | CC | Included in Toporcov, 2015 [50] |
| Huang, 2019 [167] | CC | Included in Yang, 2014 [168] |
| Inoue-Choi, 2019 [169] | CO | Included in Coleman, 2020 [170] |
| Lubin, 2010 [171] | PA | Included in Toporcov, 2015 [50] and Marron, 2010 [172] |
| Muscat, 1996 [173] | CC | Included in Kabat, 1994 [174] and [50] |
| Negri, 1993 [175] | CC | Included in La Vecchia, 1999 [155] |
| Peters, 2005 [176] | CC | Included in Applebaum, 2007 [177] |
| Radoi, 2013 [178] | CC | Included in Radoi, 2013 [179] |
| Sanchez, 2003 [180] | CC | Included in Toporcov, 2015 [50] |
| Schlecht, 1999 [181] | CC | Same subjects of Schlecht, 1999 [182] |
| Xie, 2004 [183] | CC | Included in Hayes, 1999 [184] and Toporcov, 2015 [50] |
| Zheng, 1997 [185] | CC | Included in Zheng, 1990 [186] |

CC: case-control study; CO: cohort study; NA: not available; PA: pooled-analysis.

**Supplementary Table 3.** Main characteristics of the 87 case-control studies on the association between cigarette smoking and oral and pharyngeal cancer risk included in the review, and information contributing to the meta-analysis

| **First author, year** | **Country** | **Sex** | **Type of controls** | **N. Cases** | **N. Controls** | **Site** | **Status** | | | **Intensity** | | **Duration** | | **TSQ** |
| --- | --- | --- | --- | --- | --- | --- | --- | --- | --- | --- | --- | --- | --- | --- |
|  |  |  |  |  |  |  | **Current** | **Former** | **Ever** | **Current** | **Ever** | **Current** | **Ever** | **Former** |
| Adeyemi, 2011 [187] | Nigeria | MF | H | 69 | 69 | OC SQ |  |  | X |  |  |  |  |  |
| Anantharaman, 2016 [188] | Europe | MF | P | 1292^c^ | 1425^c^ | PH | X | X |  |  |  |  |  |  |
| Applebaum, 2007 [177] | USA | MF | P | 485^c^ | 549^c^ | OC and PH SQ |  |  |  |  |  |  |  |  |
| Balaram, 2002 [189] | India | MF | H | 591^c^ | 582^c^ | OC | X | X |  |  |  |  |  | X |
| Berthiller, 2016 [5] | Europe | MF | HP | 2364^c^ | 13416^c^ | OC and PH |  |  |  |  |  |  |  |  |
| Blot, 1988 [190] | USA | MF | P | 1114^c^ | 1268^c^ | OP |  | O |  |  |  |  | X | X |
| Bundgaard, 1995 [191] | Denmark | MF | P | 161 | 400 | OC SQ |  |  | O |  | X |  |  |  |
| Castellsague, 2004 [192] | Spain | MF | H | 375^c^ | 375^c^ | OC | X | X |  |  |  | X | X | X |
| Chandran, 2005 [193] | South Africa | MF | H | 67 | 67 | OP SQ | X | X | O |  |  |  |  |  |
| Chen, 2001 [194] | USA | MF | P | 341 | 552 | OC | X | X | O |  |  |  |  |  |
| Choi, 1991 [195] | South Korea | MF | H | 309 | 927 | OC and PH | O | O | O |  | X |  | X | X |
| Day, 1993 [196] | USA | MF | P | 1065^c^ | 1182^c^ | OC |  |  |  |  |  |  |  |  |
| De Stefani, 1994 [197] | Uruguay | M | H | 246 | 253 | OP |  |  | O |  |  |  |  |  |
| De Stefani, 2007 [160] | Uruguay | M | H | 776 | 1501 | OC and PH | O | O | O |  | X |  | X | X |
| Dikshit, 2000 [198] | India | M | P | 247 | 260 | PH |  |  | O |  | X |  |  |  |
| D'Souza, 2007 [199] | USA | MF | H | 100 | 200 | PH SQ |  |  | X |  |  |  |  |  |
| Escribano Uzcudun, 2002 [200] | Spain | MF | H | 232 | 232 | PH SQ | X | X | O |  |  |  |  |  |
| Franceschi, 1990 [201] | Italy | M | H | 291^c^ | 1272^c^ | OC and PH |  |  |  | X |  | X |  |  |
| Gallus, 2003 [164] | Italy, Switzerland | MF | H | 749^c^ | 1770^c^ | OC and PH |  |  |  | X |  | X | O |  |
| Garrote, 2001 [202] | Cuba | MF | H | 200^c^ | 200^c^ | OP | O | X |  | X |  |  |  | X |
| Ghani, 2019 [203] | Malaysia | MF | H | 790 | 450 | OC |  |  | X |  |  |  |  |  |
| Gholap, 2023 [204] | India | M | P | 558 | 893 | OC and PH |  |  | O |  |  |  |  |  |
| Gunasekera, 2015 [205] | Sri Lanka | MF | P | 78 | 51 | OP |  |  | X |  |  |  |  |  |
| Hashibe, 2009 [19] | Italy, Switzerland, USA, and Puerto Rico^b^ | MF | HP | 6774^c^ | 16168^c^ | OC and PH |  |  |  |  | O |  |  |  |
| Hayes, 1999 [184] | Puerto Rico | MF | P | 342^c^ | 521^c^ | OP | O | O |  |  |  |  |  | O |
| Horn-Ross, 1997 [206] | USA, Mexico | M | P | 150 | 191 | OC | X | X | O |  |  |  |  |  |
| Hung, 1997 [207] | Taiwan | M | P | 41 | 123 | OC |  |  | X |  |  |  |  |  |
| Ishan, 2011 [208] | India | MF | P | 116 | 282 | OC |  |  | X |  |  |  |  |  |
| Jussawalla, 1971 [209] | India | MF | P | 494 | 1853 | OC and PH |  |  | O |  |  |  |  |  |
| Kabat, 1989 [210] | USA | MF | H | 761 | 1524 | OP | O | X | X | X |  |  |  |  |
| Kabat, 1989 [211] | USA | F | H | 125 | 107 | OP | X | X | O |  | X |  |  |  |
| Kabat, 1994 [174] | USA | MF | H | 1560^c^ | 2948^c^ | OP |  | X |  |  |  |  |  | X |
| Katoh, 1999 [212] | Japan | MF | H | 92 | 147 | OC SQ |  |  | X |  |  |  |  |  |
| Ko, 1995 [213] | Taiwan | MF | H | 107 | 200 | OC | X | X | O |  |  |  |  |  |
| Kune, 1993 [214] | Australia | M | P | 41 | 398 | OP SQ | X | X | X |  |  |  |  |  |
| La Vecchia, 1990 [215] | Italy | M | H | 291^c^ | 1272^c^ | OP |  |  |  |  | X |  | X |  |
| La Vecchia, 1999 [155] | Italy, Switzerland | MF | H | 1280^c^ | 4179^c^ | OC and PH |  | O |  |  |  |  |  | X |
| Lakhanpal, 2014 [216] | India | MF | P | 125 | 207 | OC |  |  | X |  |  |  |  |  |
| Lee, 2005 [217] | Taiwan | M | H | 148^c^ | 255^c^ | PH | X | X | X |  | X |  | X |  |
| Lee, 2009 [153] | Europe | MF | HP | 974 | 2221 | OP | X | X | O |  | X |  | X |  |
| Lee, 2012 [218] | Taiwan | MF | H | 1041 | 2250 | OC and PH |  |  | O |  |  |  |  |  |
| Lee, 2019 [219] | China, Taiwan | MF | H | 610 | 806 | OC and PH |  |  | O |  |  |  |  |  |
| Lissowska, 2003 [220] | Poland | MF | H | 122^c^ | 124^c^ | OC and PH | X | X |  | X |  |  |  | X |
| Llewellyn, 2004 [221] | England | MF | P | 53 | 91 | OP SQ | X | X | X |  | X |  | X |  |
| Llewellyn, 2004 [222] | England | MF | P | 116 | 207 | OP SQ | X | X | X |  | X |  | X |  |
| Madani, 2010 [223] | India | MF | P | 350 | 350 | OC |  |  | X |  |  |  |  |  |
| Mahapatra, 2015 [224] | India | MF | H | 134 | 268 | OC |  |  | X |  |  |  |  |  |
| Marron, 2010 [172] | Europe | MF | HP | 7655^c^ | 21383^c^ | OC and PH | O | O |  |  |  |  |  | X |
| Marques, 2008 [225] | Brazil | MF | H | 309 | 468 | OC and PH | X | X | O |  |  |  |  |  |
| Marshall, 1992 [226] | USA | MF | P | 290 | 290 | OP |  |  | O |  |  |  |  |  |
| Martinez, 1969 [227] | Puerto Rico | MF | P | 400 | 1200 | OC and PH |  |  | O |  | X |  |  |  |
| Merletti, 1989 [228] | Italy | MF | P | 122 | 606 | OC and PH SQ | O |  |  | X |  | X |  | X |
| Moreno-Lopez, 2000 [229] | Spain | MF | H | 75 | 150 | OP | X | X | O |  |  |  |  |  |
| Maurya, 2015 [230] | India | MF | P | 750 | 750 | OC and PH |  |  | O |  |  |  |  |  |
| Naghibzadeh-Tahami, 2024 [231] | Iran | MF | P | 133 | 266 | OC | X | X | X |  | X |  | X |  |
| Nandakumar, 1990 [232] | India | MF | P | 348 | 348 | OC |  |  | X |  |  |  |  |  |
| Oudjehih, 2020 [233] | Algeria | MF | HP | 192 | 384 | OC |  |  | O |  |  |  |  |  |
| Radoi, 2013 [179] | France | MF | P | 772 | 3555 | OC | X | O | O | O | O | O | O | X |
| Radoi, 2015 [234] | France | MF | P | 689 | 3481 | OP SQ |  |  |  |  |  |  |  |  |
| Rodriguez, 2004 [235] | Italy, Switzerland | MF | H | 137^c^ | 298^c^ | OP |  |  |  | X |  |  | X |  |
| Sankaranarayanan, 1989 [236] | India | M | H | 158 | 314 | OC |  |  | O |  |  |  | X |  |
| Sapkota, 2007 [237] | India | MF | HP | 513 | 718 | PH |  |  | O |  | X |  | X |  |
| Schildt, 1998 [238] | Sweden | MF | P | 410 | 410 | OC SQ | X | X | X |  |  |  |  |  |
| Schlecht, 1999 [182] | Brazil | MF | H | 590 | 1578 | OC and PH | O | O | O |  |  |  |  | X |
| Shewale, 2021 [239] | USA | MF | H | 249 | 498 | OP SQ | X | X | O |  | X |  | X | X |
| Smith, 1998 [240] | USA | MF | H | 93^c^ | 205^c^ | OP |  | X |  |  |  |  |  |  |
| Smith, 2010 [241] | USA | MF | H | 201 | 324 | OC and PH |  |  | O |  |  |  |  |  |
| Su, 1998 [242] | China | M | H | 62 | 62 | OC |  |  | X |  | X |  | X |  |
| Subapriya, 2007 [243] | India | MF | P | 304 | 366 | OC SQ | X |  |  |  |  |  |  |  |
| Sugimura, 2006 [244] | Japan | MF | H | 122 | 241 | OC SQ |  |  | X |  |  |  |  |  |
| Szymanska, 2011 [245] | Brazil, Argentina, Cuba | MF | H | 1030^c^ | 1707^c^ | OP |  | X |  |  |  |  | X | X |
| Takezaki, 1996 [246] | Japan | MF | H | 266 | 36527 | OP | X | O | O | X |  | X |  | X |
| Takezaki, 2000 [247] | Japan | M | H | 62^c^ | 11936^c^ | PH |  |  |  |  |  |  |  |  |
| Toporcov, 2015 [50] | Europe, USA, and Puerto Rico^b^ | MF | HP | 9946 | 25976 | OC and PH |  |  | O |  |  |  |  |  |
| Tuyns, 1988 [248] | Italy, Spain, Switzerland, and France^b^ | M | P | 281 | 3057 | PH |  |  | O |  | X |  |  |  |
| Varela-Lema, 2010 [249] | Spain | MF | H | 92 | 236 | OP | X | X | X | X | X | X | X | X |
| Vlajinac, 2006 [250] | Serbia, Montenegro | MF | H | 100 | 100 | PH | X | X | O |  |  |  |  |  |
| Wang, 2005 [251] | USA | MF | H | 348^c^ | 330^c^ | OP SQ |  |  |  |  |  |  |  |  |
| Wasnik, 1998 [252] | India | MF | H | 123 | 246 | PH |  |  | X |  |  |  |  |  |
| Werbrouck, 2008 [253] | Belgium | MF | P | 102 | 157 | OC and PH SQ |  |  | O |  |  |  |  |  |
| Winn, 1981 [254] | USA | F | H | 255 | 502 | OC and PH |  |  | O |  | X |  |  |  |
| Yang, 2014 [168] | Taiwan | MF | HP | 592 | 623 | OC and PH SQ |  |  | O |  |  |  |  |  |
| Yang, 2023 [255] | USA | MF | HP | 377 | 433 | OC and PH SQ |  |  | X |  |  |  |  |  |
| Zavras, 2001 [256] | Greece | MF | H | 110 | 115 | OP | X | X | O |  |  |  |  |  |
| Zeng, 2019 [257] | China | M | H | 278 | 767 | PH |  |  | X |  | X |  | X | X |
| Zheng, 1990 [186] | China | MF | H | 404 | 404 | OC | X | X | X |  | X |  | X |  |
| Znaor, 2003 [258] | India | M | HP | 2199 | 3638 | OC and PH | O | O | O |  | X |  | X | X |
| **Total (1969-2023)^b^** |  |  |  | **30540** | **102500** |  | **36** | **38** | **62** | **10** | **23** | **7** | **22** | **19** |

F: females; H: hospital; M: males; OC: oral cavity; OP: oral cavity and pharynx, no distinction is made; P: population; PH: pharynx; SQ: squamous cell carcinoma; TSQ: time-since-quitting; X symbol indicates that estimates were provided in the original study publication; O symbol indicates that estimates were derived from the information provided in the original study publication.

^a^ Pooled-analysis; ^b^ For status, intensity, duration, and TSQ, numbers represent the number of studies providing information; ^c^ Number of subjects not included in the total, because overall estimates are already included in other articles.

**Supplementary Table 4.** Main characteristics of the 28 cohort studies on the association between cigarette smoking and oral and pharyngeal cancer risk included in the review and corresponding information contributing to the meta-analysis.

| **First author, year** | **Country** | **Sex** | **Endpoint** | **NCases** | **Site** | **Status** | | | **Intensity** | | **Duration** | | **TSQ** |
| --- | --- | --- | --- | --- | --- | --- | --- | --- | --- | --- | --- | --- | --- |
|  |  |  |  |  |  | **Current** | **Former** | **Ever** | **Current** | **Ever** | **Current** | **Ever** | **Former** |
| Agudo, 2012 [259] | Europe (EPIC) | MF | i | 553 | OC and PH | X | X | O |  |  |  |  |  |
| Akiba, 1990 [260] | Japan (6-pref) | MF | m | 112 | OC | X |  |  | X |  |  |  |  |
| Akiba, 1994 [261] | Japan (LSS) | MF | i | 69 | PH |  | X |  |  |  |  |  |  |
| Ansary-Moghadda, 2009 [2] | Australia and Asia (APCSC)^a^ | MF | m | 134 | OP | O |  |  | X |  |  |  |  |
| Blakely, 2013 [262] | New Zeland (NZCR) | MF | i | 1815 | OP | X | X | O |  |  |  |  |  |
| Carter, 2015 [6] | USA (multiple cohorts)^b^ | MF | m | 245 | OC | X | X | O |  |  |  |  |  |
| Christensen, 2018 [263] | USA (NLMS) | MF | m | 160 | OP | X | X | O |  |  |  |  |  |
| Coleman, 2020 [170] | USA (NHIS) | MF | m | 374 | OP | X | X | O |  |  |  |  |  |
| Freedman, 2007 [264] | USA (NIH-AARP) | MF | i | 449^c^ | OC and PH |  | O | O | X | X |  |  | X |
| Freedman, 2016 [265] | USA (NIH-AARP) | MF | i | 546 | OC and PH | O |  |  | X |  |  |  |  |
| Friborg, 2007 [266] | Singapore (SCHS) | MF | i | 75 | PH | X | X | O | X |  | X |  | X |
| Hsu, 2014 [267] | Taiwan (SCHIP, MRFMD and CBCSP)^b^ | M | i |  | OC and PH |  |  | O |  | O |  | O |  |
| Ide, 2008 [268] | Japan (JACC) | MF | i | 52^c^ | OP |  |  |  | X |  |  |  |  |
| Jacob, 2018 [269] | UK (GPO) | MF | i |  | OP |  |  | X |  |  |  |  |  |
| Katanoda, 2008 [26] | Japan (JPHC, 3-pref and JACC)^b^ | MF | m |  | OP | X | X | X |  |  |  |  |  |
| Liaw, 1998 [270] | Taiwan (12-town) | MF | m | 13^c^ | OP |  |  |  |  |  | X |  |  |
| Lu, 2018 [271] | Japan (JPHC) | MF | i | 222^c^ | PH |  |  |  |  |  |  |  |  |
| Maasland, 2014 [272] | Netherlands (NCS) | MF | i | 193 | OC and PH | O | O | O |  | X |  | X | X |
| McLaughlin, 1995 [273] | USA (BIRLS) | MF | m | 332 | OC and PH | O | O | O | X |  |  |  |  |
| Mezzoiuso, 2021 [274] | Italy (FRiCaM) | F | i | 112 | OC | X | X | O |  |  |  |  |  |
| Morales, 2020 [275] | UK (THIN) | MF | i | 3516 | OC | O | O | O |  |  |  |  |  |
| Muwonge, 2008 [276] | India (TOCS) | MF | i | 282 | OC |  |  | X |  |  |  |  |  |
| Nordlund, 1997 [277] | Sweden (SCR) | F | i | 63 | OC and PH | O |  |  |  |  |  |  |  |
| Offermans, 2014 [278] | Netherlands(NCS) | M | i | 113^c^ | OC and PH |  |  |  |  |  |  |  |  |
| Pednekar, 2011 [279] | India (MCR) | M | i | 287 | OP |  |  | X |  |  |  |  |  |
| Saito, 2013 [280] | Japan (IPHS) | MF | m | 94 | PH | X | X | O | X |  |  |  |  |
| Wen, 2004 [281] | Taiwan (TOS and MPTNCST)^b^ | M | m | 69 | OP | X |  |  | X |  |  |  |  |
| Yun, 2005 [282] | South Korea (NHIC) | M | i | 172 | OP | X | X | O | X |  | X | O |  |
| **Total (1990-2021)^a^** |  |  |  | **9203** |  | **18** | **15** | **18** | **10** | **3** | **3** | **3** | **3** |

12: 12 Townships Study; 3-pref: 3 Prefectures Study; 6-pref: 6 prefectures Study; APCSC: Asian Pacific Cohort Study Collaboration; ARCAGE: European Alcohol Related Cancers and Genetic susceptibility in Europe; BIRLS: Beneficiary Identification and Records Locator Subsystem of the Veteran Administration; CBCSP: Community-based Cancer Screening Project cohort; EPIC: European Prospective Investigation into Cancer and Nutrition; F: females; FRiCaM: Fattori di Rischio per il Carcinoma della Mammella Cohort; GPO: General Practitioners’ offices; i: incidence; IPHS: Ibaraki Prefectural Health Study; JACC: Japan Collaborative Cohort Study; JPHC: Japan Public Health Center-based Prospective Study; LSS: Life Span Study; m:mortality; M: males; MCR: Mumbai Cancer Registry; MPTNCST: Metropolitan precincts and urban and rural townships in northern, central, and southern Taiwan; MRFMD: Multiple Risk Factors for Multiple diseases cohort; NCS: Netherlands Cohort Study; NHIC: National Health Insurance Corporation; NHIS: Public national Health Interview Survey; NIH-AARP: National Institutes of Health/American Association of Retired Persons; NLMS: National Lifetime Mortality Study; NZCR: New Zealand Cancer Registry; OC: oral cavity; OP: oral cavity and pharynx, no distinction is made; PH: pharynx; SCHIP: Six-community Hypertension Intervention Project cohort; SCHS: Singapore Chinese Health Study; SCR: Sweden Cancer Registry; THIN: The Health Improvement Network; TOCS: Trivandrum Oral Cancer Screening Study; TOS: Taipei Outpatient Service Center; TSQ: time-since-quitting; X symbol indicates that estimates were provided in the original study publication; O symbol indicates that estimates were derived from the information provided in the original study publication.

^a^ Pooled-analysis; ^b^ For status, intensity, duration, and TSQ, numbers represent the number of studies providing information; ^c^ Number of subjects not included in the total, because overall estimates are already included in other articles.

**Supplementary Table 5.** List of publications containing data that was partially excluded from the meta-analysis and reason for exclusion.

| **First author (year)** | **Excluded estimate** | **Reason for exclusion** |
| --- | --- | --- |
| Anantharaman, 2016 [188] | Status (ever vs never, also for pharynx), intensity | Included in Lee, 2009 [153] and Agudo, 2012 [259] |
| Applebaum, 2007 [177] | Status (ever vs never, also for oral cavity and pharynx) | Included in Toporcov, 2015 [50] |
| Balaram, 2002 [189] | Status (ever vs never, also for oral cavity), time since quitting | Included in Herrero, 2003 [166] |
| Berthiller, 2016 [5] | Status (ever vs never) and intensity for oral cavity | Included in Toporcov, 2015 [50] |
| Blot, 1988 [190] | Status (current vs never and ever vs never), intensity | Included in Toporcov, 2015 [50] |
| Castellsague, 2004 [192] | Status (ever vs never, also for pharynx), intensity | Included in Herrero, 2003 [166] |
| Day, 1993 [196] | Status (current vs never also for oral cavity, former vs never, ever vs never also for oral cavity), intensity, duration, and time since quitting | Included in Blot, 1988 [190] and Toporcov, 2015 [50] |
| Franceschi, 1990 [201] | Status (current vs never, former vs never, ever vs never, also for oral cavity and pharynx), time since quitting | Included in La Vecchia, 1999 [155] and Toporcov, 2015 [50] |
| Freedman, 2007 [264] | Status (currnev, also for oral cavity and pharynx) | Included in Freedman, 2016 [265] |
| Gallus, 2003 [164] | Status (current vs never also for oral cavity, former vs never, ever vs never also for oral cavity), time since quitting | Included in La Vecchia, 1999 [155] |
| Garrote, 2001 [202] | Status (ever vs never) | Included in Herrero, 2003 [166] |
| Hashibe, 2009 [19] | Status (ever vs never, also for oral cavity and pharynx) | Included in Toporcov, 2015 [50] |
| Hayes, 1999 [184] | Status (ever vs never), intensity | Included in Toporcov, 2015 [50] |
| Ide, 2008 [268] | Status (current vs never, former vs never, ever vs never) | Included in Katanoda, 2008 [26] |
| Kabat, 1994 [174] | Status (current vs never, ever vs never) | Included in Toporcov, 2015 [50] |
| La Vecchia, 1990 [215] | Status (current vs never, former vs never, ever vs never), intensity, time since quitting | Included in La Vecchia, 1999 [155] |
| La Vecchia, 1999 [155] | Status (current vs never, ever vs never, also for oral cavity and pharynx) | Included in Toporcov, 2015 [50] |
| Liaw, 1998 [270] | Status (current vs never), intensity | Included in Wen, 2004 [281] and Hsu, 2014 [267] |
| Lissowska, 2003 [220] | Status (ever vs never) | Included in Herrero, 2003 [166] |
| Lu, 2018 [271] | Status (current vs never, former vs never and ever vs never) for total oral cavity and pharynx | Included in Katanoda, 2008 [26] |
| Marron, 2010 [172] | Status (ever vs never) | Included in Toporcov, 2015 [50] |
| Radoi, 2015 [234] | Status (ever vs never) | Included in Radoi, 2013 [179] |
| Rodriguez, 2004 [235] | Status (current vs never, former vs never and ever vs never), time since quitting | Included in La Vecchia, 1999 [155] |
| Smith, 1998 [240] | Status (current vs never and ever vs never) | Included in Toporcov, 2015 [50] |
| Szymanska, 2011 [245] | Status (current vs never and ever vs never), intensity | Included in Toporcov, 2015 [50] |
| Takezaki, 2000 [247] | Status (current vs never, former vs never and ever vs never) and time since quitting for oral and pharynx | Included in Takezaki, 1996 [246] |
| Wang, 2005 [251] | Status (current vs never and ever vs never) | Included in Toporcov, 2015 [50] |

**Supplementary Table 6.** Quality evaluation of the 87 case-control studies included in the present meta-analysis using the New-Castle Ottawa (NOS) scale.

| **Author, Year** | **SELECTION** | | | | **COMPARABLITY** | **EXPOSURE** | | | **TOTAL NOS SCORE** |
| --- | --- | --- | --- | --- | --- | --- | --- | --- | --- |
|  | Adequate definition of cases | Representiveness of cases | Selection of Controls | Definition of controls | Comparability of cases and controls^b^ | Ascertainment of exposure | Same methods of ascertainment of exposure | Non-response rate |  |
| Adeyemi, 2011 [187] | ☆ | ☆ | - | - | ☆ | ☆ | ☆ | - | 5 |
| Anantharaman, 2016 [188] | ☆ | - | ☆ | - | ☆☆ | - | ☆ | - | 5 |
| Applebaum, 2007 [177] | ☆ | ☆ | ☆ | - | ☆☆ | - | ☆ | - | 6 |
| Balaram, 2002 [189] | ☆ | ☆ | - | - | ☆☆ | - | ☆ | ☆ | 6 |
| Berthiller, 2016 [5] | ☆ | - | - | - | ☆☆ | - | ☆ | - | 4 |
| Blot, 1988 [190] | ☆ | ☆ | ☆ | - | ☆☆ | - | ☆ | ☆ | 7 |
| Bundgaard, 1995 [191] | ☆ | ☆ | ☆ | - | ☆☆ | - | ☆ | - | 6 |
| Castellsague, 2004 [192] | ☆ | ☆ | - | - | ☆☆ | ☆ | ☆ | - | 6 |
| Chandran, 2005 [193] | ☆ | - | - | ☆ | ☆ | - | ☆ | ☆ | 5 |
| Chen, 2001 [194] | ☆ | - | ☆ | - | ☆☆ | - | ☆ | ☆ | 6 |
| Choi, 1991 [195] | ☆ | - | - | ☆ | ☆☆ | ☆ | ☆ | - | 6 |
| Day, 1993 [196] | ☆ | ☆ | ☆ | - | ☆☆ | - | ☆ | ☆ | 7 |
| De Stefani, 1994 [197] | ☆ | ☆ | - | - | ☆☆ | - | ☆ | ☆ | 6 |
| De Stefani, 2007 [160] | ☆ | ☆ | - | - | ☆☆ | - | ☆ | ☆ | 6 |
| Dikshit, 2000 [198] | ☆ | - | ☆ | - | ☆☆ | - | ☆ | - | 5 |
| D'Souza, 2007 [199] | ☆ | - | - | ☆ | - | - | ☆ | ☆ | 3 |
| Escribano Uzcudun, 2002 [200] | ☆ | ☆ | - | ☆ | ☆☆ | - | ☆ | ☆ | 7 |
| Franceschi, 1990 [201] | ☆ | - | - | - | ☆☆ | - | ☆ | - | 4 |
| Gallus, 2003 [164] | ☆ | - | - | - | ☆☆ | - | ☆ | ☆ | 5 |
| Garrote, 2001 [202] | ☆ | ☆ | - | - | ☆☆ | - | ☆ | ☆ | 6 |
| Ghani, 2019 [203] | ☆ | - | - | - | ☆ | - | ☆ | - | 3 |
| Gholap, 2023 [204] | ☆ | ☆ | ☆ | - | ☆☆ | - | ☆ | - | 6 |
| Gunasekera, 2015 [205] | ☆ | ☆ | ☆ | ☆ | ☆☆ | - | ☆ | - | 7 |
| Hashibe, 2009 [19] | ☆ | - | - | - | ☆☆ | - | ☆ | - | 4 |
| Hayes, 1999 [184] | ☆ | ☆ | ☆ | - | ☆☆ | - | ☆ | - | 6 |
| Horn-Ross, 1997 [206] | ☆ | ☆ | ☆ | - | ☆ | - | ☆ | ☆ | 6 |
| Hung, 1997 [207] | ☆ | ☆ | ☆ | - | ☆☆ | - | ☆ | - | 6 |
| Ishan, 2011 [208] | ☆ | ☆ | ☆ | - | ☆☆ | - | ☆ | - | 6 |
| Jussawalla, 1971 [209] | ☆ | ☆ | ☆ | - | - | - | ☆ | - | 4 |
| Kabat, 1989 [210] | ☆ | - | - | - | ☆☆ | - | ☆ | - | 4 |
| Kabat, 1989 [211] | ☆ | - | - | - | ☆☆ | - | ☆ | - | 4 |
| Kabat, 1994 [174] | ☆ | ☆ | - | ☆ | ☆☆ | - | ☆ | - | 6 |
| Katoh, 1999 [212] | ☆ | - | - | - | ☆☆ | - | ☆ | - | 4 |
| Ko, 1995 [213] | ☆ | ☆ | - | - | ☆☆ | - | ☆ | - | 5 |
| Kune, 1993 [214] | ☆ | ☆ | ☆ | - | ☆☆ | - | ☆ | - | 6 |
| La Vecchia, 1990 [215] | ☆ | - | - | - | ☆☆ | - | ☆ | ☆ | 5 |
| La Vecchia, 1999 [155] | - | - | - | - | ☆☆ | - | ☆ | - | 3 |
| Lakhanpal, 2014 [216] | ☆ | - | ☆ | - | ☆☆ | - | ☆ | - | 5 |
| Lee, 2005 [217] | ☆ | ☆ | - | - | ☆☆ | - | ☆ | - | 5 |
| Lee, 2009 [153] | ☆ | ☆ | - | - | ☆☆ | - | ☆ | ☆ | 6 |
| Lee, 2012 [218] | ☆ | ☆ | - | - | ☆☆ | - | ☆ | - | 5 |
| Lee, 2019 [219] | ☆ | ☆ | - | - | ☆☆ | - | ☆ | ☆ | 6 |
| Lissowska, 2003 [220] | ☆ | ☆ | - | - | ☆☆ | - | ☆ | - | 4 |
| Llewellyn, 2004 [221] | ☆ | ☆ | ☆ | ☆ | ☆☆ | - | ☆ | - | 6 |
| Llewellyn, 2004 [222] | ☆ | ☆ | ☆ | ☆ | ☆☆ | - | ☆ | - | 6 |
| Madani, 2010 [223] | ☆ | ☆ | ☆ | ☆ | ☆☆ | - | ☆ | - | 6 |
| Mahapatra, 2015 [224] | ☆ | ☆ | - | ☆ | - | - | ☆ | - | 4 |
| Marques, 2008 [225] | ☆ | ☆ | - | ☆ | ☆☆ | - | ☆ | - | 5 |
| Marron, 2010 [172] | ☆ | - | - | - | ☆ | - | ☆ | - | 3 |
| Marshall, 1992 [226] | ☆ | ☆ | ☆ | - | ☆ | - | ☆ | - | 4 |
| Martinez, 1969 [227] | ☆ | ☆ | ☆ | - | ☆ | - | ☆ | - | 4 |
| Merletti, 1989 [228] | ☆ | ☆ | ☆ | - | ☆ | - | ☆ | ☆ | 5 |
| Moreno-Lopez, 2000 [229] | ☆ | ☆ | ☆ | ☆ | - | - | ☆ | - | 5 |
| Mauraya, 2015 [230] | ☆ | - | ☆ | ☆ | - | - | ☆ | - | 4 |
| Naghibzadeh -Tahami, 2024 [231] | ☆ | ☆ | ☆ | ☆ | ☆☆ | - | ☆ | - | 7 |
| Nandakumar, 1990 [232] | ☆ | ☆ | - | - | ☆ | - | ☆ | - | 3 |
| Oudjehih, 2020 [233] | ☆ | ☆ | - | - | ☆☆ | - | ☆ | - | 4 |
| Radoi, 2013 [179] | ☆ | ☆ | ☆ | - | ☆☆ | - | ☆ | ☆ | 6 |
| Radoi, 2015 [234] | ☆ | ☆ | ☆ | - | ☆☆ | - | ☆ | ☆ | 7 |
| Rodriguez, 2004 [235] | ☆ | - | - | - | ☆☆ | - | ☆ | - | 4 |
| Sankaranarayanan, 1989 [236] | ☆ | ☆ | - | - | ☆ | - | ☆ | - | 3 |
| Sapkota, 2007 [237] | - | - | ☆ | - | ☆☆ | - | ☆ | - | 4 |
| Schildt, 1998 [238] | ☆ | ☆ | ☆ | - | ☆ | - | ☆ | - | 4 |
| Schlecht, 1999 [182] | ☆ | ☆ | - | - | ☆ | ☆ | ☆ | - | 4 |
| Shewale, 2021 [239] | - | - | - | ☆ | ☆ | - | ☆ | - | 2 |
| Smith, 1998 [240] | ☆ | ☆ | - | ☆ | ☆☆ | - | ☆ | ☆ | 7 |
| Smith, 2010 [241] | ☆ | ☆ | - | ☆ | ☆☆ | - | ☆ | ☆ | 7 |
| Su, 1998 [242] | ☆ | - | - | - | ☆☆ | - | ☆ | - | 3 |
| Subapriya, 2007 [243] | ☆ | ☆ | - | - | ☆☆ | - | ☆ | ☆ | 6 |
| Sugimura, 2006 [244] | ☆ | ☆ | - | ☆ | ☆ | - | ☆ | - | 4 |
| Szymanska, 2011 [245] | ☆ | - | - | - | ☆☆ | - | ☆ | - | 4 |
| Takezaki, 1996 [246] | ☆ | ☆ | - | ☆ | ☆☆ | - | ☆ | ☆ | 7 |
| Takezaki, 2000 [247] | ☆ | ☆ | - | ☆ | ☆☆ | - | ☆ | - | 6 |
| Toporcov, 2015 [50] | ☆ | - | - | - | ☆☆ | - | ☆ | - | 4 |
| Tuyns, 1988 [248] | ☆ | - | ☆ | - | ☆☆ | - | ☆ | - | 5 |
| Varela-Lema, 2010 [249] | ☆ | ☆ | - | ☆ | ☆☆ | - | ☆ | ☆ | 7 |
| Vlajinac, 2006 [250] | ☆ | ☆ | - | - | ☆☆ | - | ☆ | - | 5 |
| Wang, 2005 [251] | ☆ | ☆ | - | ☆ | - | - | ☆ | ☆ | 5 |
| Wasnik, 1998 [252] | ☆ | ☆ | - | - | - | - | ☆ | - | 3 |
| Werbrouck, 2008 [253] | ☆ | ☆ | ☆ | - | ☆ | - | ☆ | - | 4 |
| Winn, 1981 [254] | ☆ | - | - | ☆ | ☆ | - | ☆ | ☆ | 4 |
| Yang, 2014 [168] | ☆ | ☆ | - | ☆ | - | - | ☆ | - | 4 |
| Yang, 2023 [255] | ☆ | ☆ | - | ☆ | ☆☆ | - | ☆ | ☆ | 7 |
| Zavras, 2001 [256] | ☆ | ☆ | - | ☆ | ☆☆ | - | ☆ | ☆ | 7 |
| Zeng, 2019 [257] | ☆ | ☆ | - | - | ☆☆ | - | ☆ | ☆ | 6 |
| Zheng, 1990 [186] | ☆ | - | - | - | ☆☆ | - | ☆ | - | 4 |
| Znaor, 2003 [258] | ☆ | ☆ | ☆ | - | ☆☆ | - | ☆ | - | 6 |

^a^ Each item could be scored with a maximum of one star, except for the item “Comparability of cases and controls” which could receive a maximum of two stars; ^b^ Studies controlling for age and sex in the design or in the analysis received one star. Studies with all the previous variables and at least one of the following variables: alcohol consumption, Human Papilloma Virus infection, chewing tobacco or betel quid, diet, and family history of head and neck cancer received two stars.

**Supplementary Table 7.** Quality evaluation of the 28 cohort studies included in the present meta-analysis using the New-Castle Ottawa (NOS) scale.

| **Author, Year** | **SELECTION** | | | | **COMPARABLITY** | **EXPOSURE** | | | **TOTAL NOS SCORE** |
| --- | --- | --- | --- | --- | --- | --- | --- | --- | --- |
|  | Representativeness of the exposed cohort | Selection of the non-exposed cohort | Ascertainment of exposure | Outcome of interest not present at start of study | Comparability of cohorts^b^ | Ascertainment of outcome | Follow-up long enough for outcome to occur^c^ | Adequacy of follow-up cohorts^d^ |  |
| Agudo, 2012 [259] | ☆ | ☆ | ☆ | ☆ | ☆☆ | ☆ | ☆ | ☆ | 9 |
| Akiba, 1990 [260] | ☆ | ☆ | ☆ | - | ☆☆ | ☆ | ☆ | - | 7 |
| Akiba, 1994 [261] | - | ☆ | ☆ | - | - | ☆ | ☆ | - | 4 |
| Ansary-Moghadda, 2009 [2] | ☆ | ☆ | - | - | ☆☆ | - | - | - | 4 |
| Blakely, 2013 [262] | ☆ | ☆ | ☆ | - | ☆ | ☆ | - | ☆ | 6 |
| Carter, 2015 [6] | - | ☆ | ☆ | - | ☆☆ | ☆ | ☆ | ☆ | 7 |
| Christensen, 2018 [263] | ☆ | ☆ | - | - | ☆ | ☆ | ☆ | - | 5 |
| Coleman, 2020 [170] | ☆ | ☆ | ☆ | - | ☆ | ☆ | ☆ | - | 6 |
| Freedman, 2007 [264] | ☆ | ☆ | ☆ | ☆ | ☆☆ | ☆ | - | - | 7 |
| Freedman, 2016 [265] | ☆ | ☆ | ☆ | ☆ | ☆☆ | ☆ | - | - | 7 |
| Friborg, 2007 [266] | ☆ | ☆ | ☆ | ☆ | ☆☆ | ☆ | ☆ | ☆ | 9 |
| Hsu, 2014 [267] | ☆ | ☆ | ☆ | - | ☆☆ | ☆ | ☆ | - | 7 |
| Ide, 2008 [268] | ☆ | ☆ | ☆ | ☆ | ☆☆ | ☆ | ☆ | - | 8 |
| Jacob, 2018 [269] | ☆ | ☆ | ☆ | ☆ | - | ☆ | ☆ | - | 6 |
| Katanoda, 2008 [26] | ☆ | ☆ | ☆ | - | ☆ | ☆ | ☆ | - | 6 |
| Liaw, 1998 [270] | ☆ | ☆ | ☆ | ☆ | ☆☆ | ☆ | ☆ | - | 8 |
| Lu, 2018 [271] | ☆ | ☆ | ☆ | - | ☆☆ | ☆ | ☆ | - | 7 |
| Maasland, 2014 [272] | ☆ | ☆ | ☆ | ☆ | ☆☆ | ☆ | ☆ | - | 8 |
| McLaughlin, 1995 [273] | - | ☆ | - | - | - | ☆ | ☆ | - | 3 |
| Mezzoiuso, 2016 [274] | ☆ | ☆ | ☆ | - | ☆ | ☆ | ☆ | - | 6 |
| Morales, 2020 [275] | ☆ | ☆ | - | ☆ | ☆☆ | ☆ | ☆ | - | 7 |
| Muwonge, 2008 [276] | ☆ | ☆ | ☆ | ☆ | - | - | - | - | 4 |
| Nordlund, 1997 [277] | ☆ | ☆ | ☆ | - | - | ☆ | ☆ | ☆ | 6 |
| Offermans, 2014 [278] | ☆ | ☆ | ☆ | ☆ | ☆☆ | ☆ | ☆ | ☆ | 9 |
| Pednekar, 2011 [279] | ☆ | ☆ | ☆ | - | ☆ | ☆ | - | - | 5 |
| Saito, 2013 [280] | ☆ | ☆ | ☆ | - | ☆☆ | ☆ | ☆ | - | 7 |
| Wen, 2004 [281] | ☆ | ☆ | ☆ | - | ☆ | ☆ | - | - | 5 |
| Yun, 2005 [282] | - | ☆ | ☆ | - | ☆☆ | ☆ | - | - | 5 |

^a^ Each item could be scored with a maximum of one star, except for the item “Comparability of cases and controls” which could receive a maximum of two stars; ^b^ Studies controlling for age and sex in the design or in the analysis received one star. Studies with all the previous variables and at least one of the following variables: alcohol consumption, Human Papilloma Virus infection, chewing tobacco or betel quid, diet, and family history of head and neck cancer received two stars; ^c^ Studies with follow-up time ≥10 years received one star; ^d^ Studies with follow-up rate ≥80% or with a description of those lost at follow-up received one star.

**Supplementary Figure 1.** Flowchart for the selection of the original studies on the association between cigarette smoking and oral and pharyngeal cancer risk included in the review and meta-analysis.


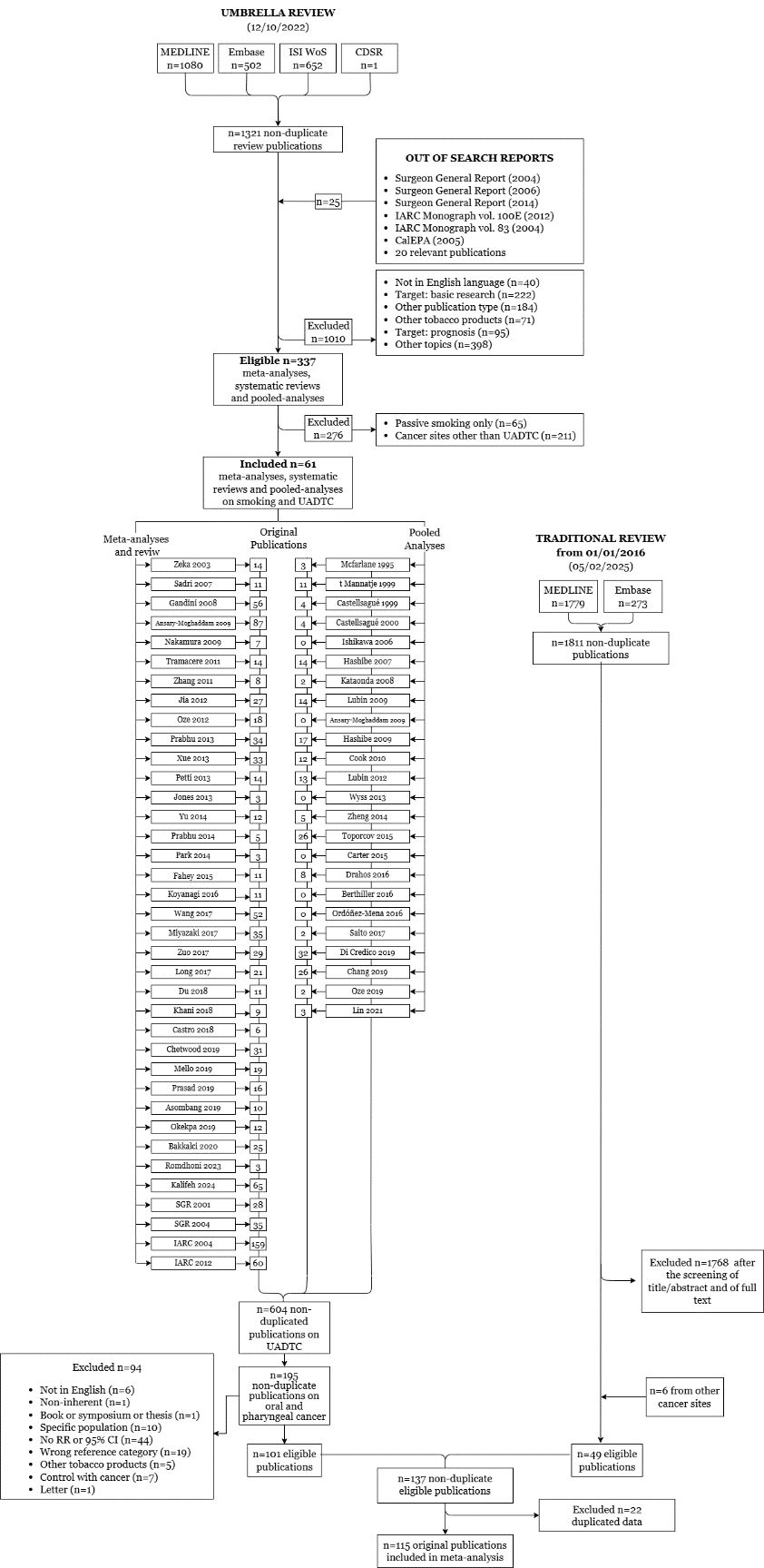


CDSR: Cochrane Database of Systematic Reviews; IC: Confidence Interval; RR: Relative Risk; SHS: second-hand smoke; UADTC: Upper Aerodigestive Tract Cancer; WoS: Web of Science.

**Supplementary Figure 2**. Forest plot of study-specific and pooled relative risk (RR) of oral and pharyngeal cancer for ever smokers (ES) versus never smokers (NS), overall and by study design.


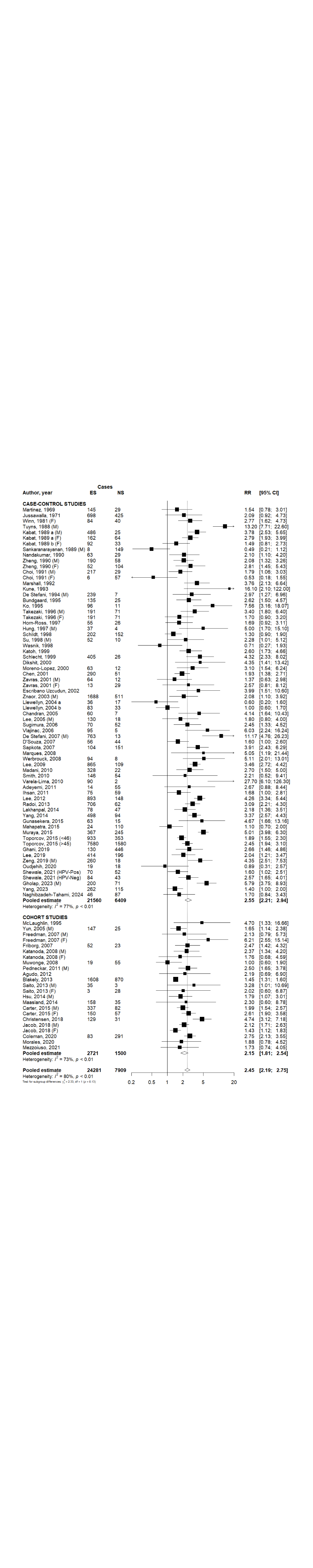


CI: confidence interval; F: females; HPV+: Human Papillomavirus test positive subjects; HPV -: Human Papillomavirus test negative subjects; M: males.

**Supplementary Figure 3**. Forest plot of study-specific and pooled relative risk (RR) of oral cancer for current smokers (CS) versus never smokers (NS), overall and by study design.


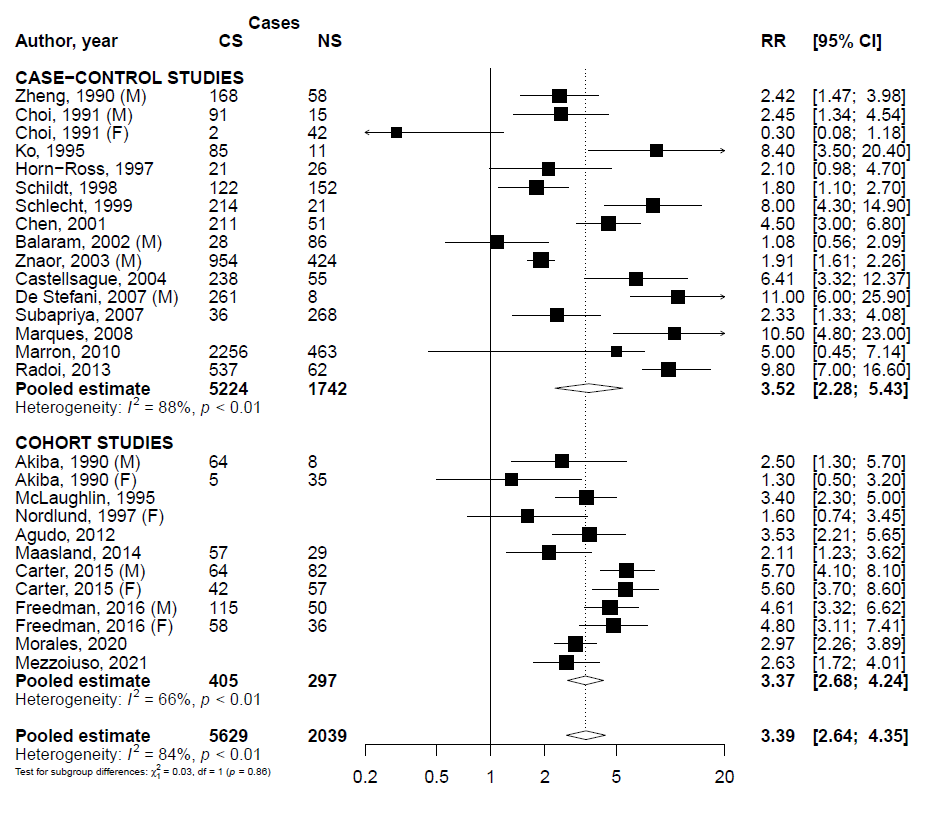


CI: confidence interval; F: females; M: males.

**Supplementary Figure 4**. Forest plot of study-specific and pooled relative risk (RR) of oral cancer for former smokers (FS) versus never smokers (NS), overall and by study design.


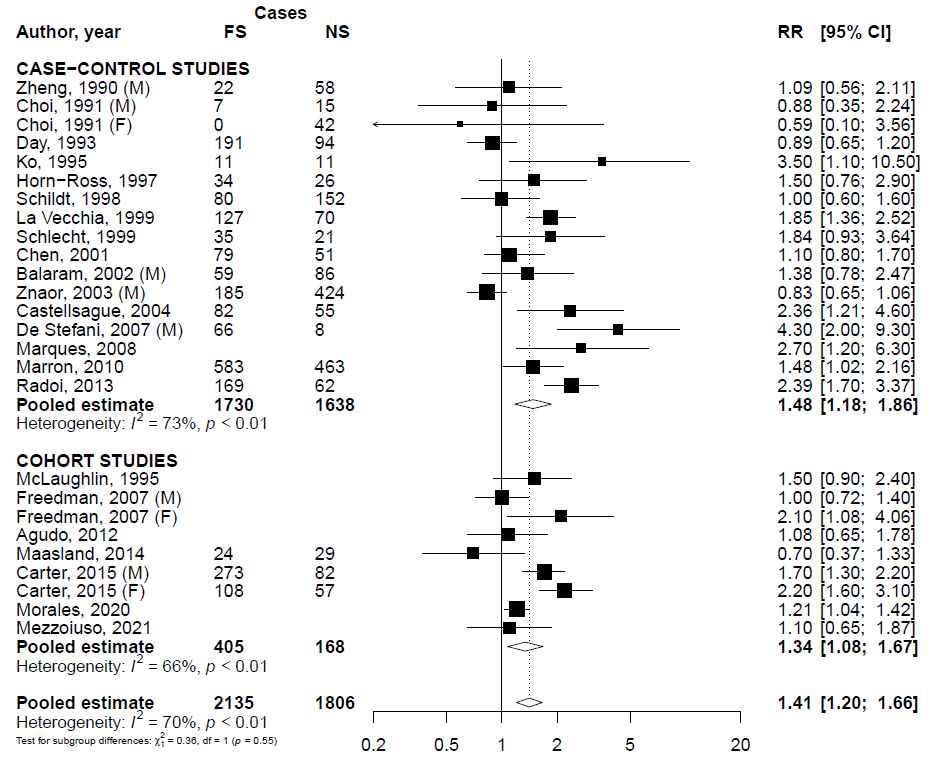


CI: confidence interval; F: females; M: males.

**Supplementary Figure 5**. Forest plot of study-specific and pooled relative risk (RR) of oral cancer for ever smokers (ES) versus never smokers (NS), overall and by study design.


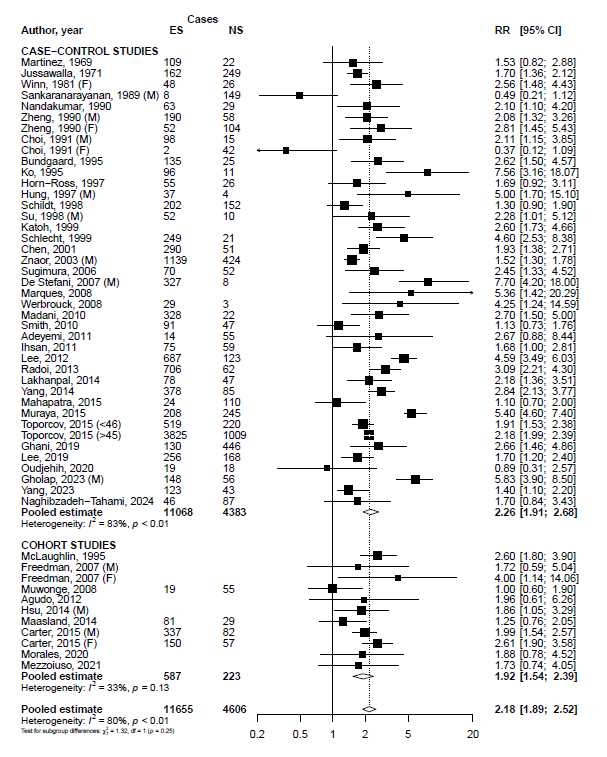


CI: confidence interval; F: females; M: males.

**Supplementary Figure 6**. Forest plot of study-specific and pooled relative risk (RR) of pharyngeal cancer for current smokers (CS) versus never smokers (NS), overall and by study design.


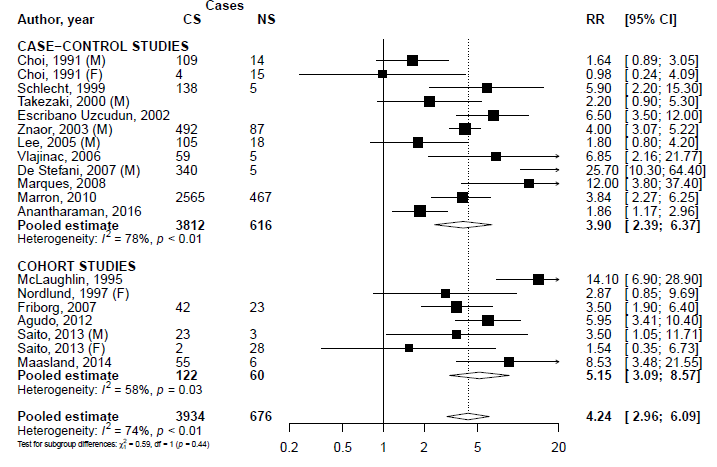


CI: confidence interval; F: females; M: males.

**Supplementary Figure 7**. Forest plot of study-specific and pooled relative risk (RR) of pharyngeal cancer for former smokers (FS) versus never smokers (NS), overall and by study design.


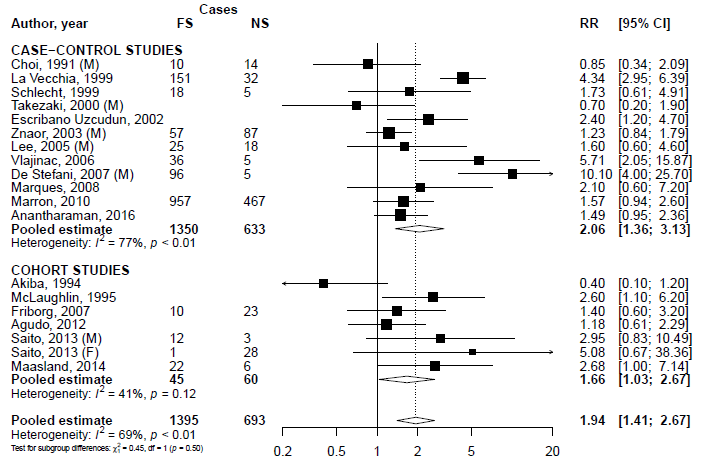


CI: confidence interval; F: females; M: males.

**Supplementary Figure 8**. Forest plot of study-specific and pooled relative risk (RR) of pharyngeal cancer for ever smokers (ES) versus never smokers (NS), overall and by study design.


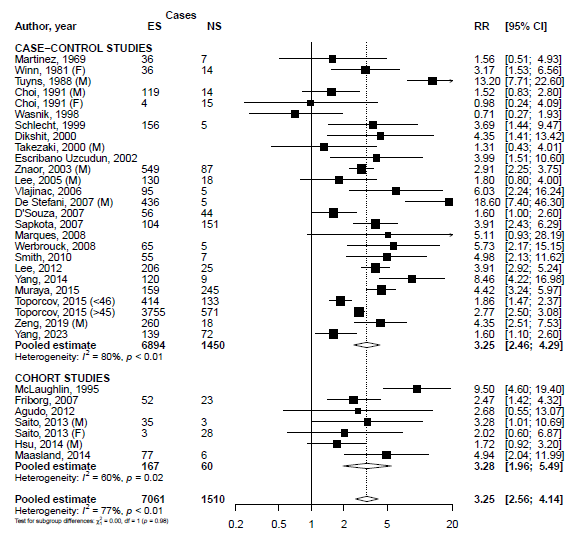


CI: confidence interval; F: females; M: males.

**Supplementary Figure 9.** Funnel plot of studies on the association between current (panel A), former (panel B), and ever (panel C) cigarette smokers versus never smokers and oral and pharyngeal cancer risk.


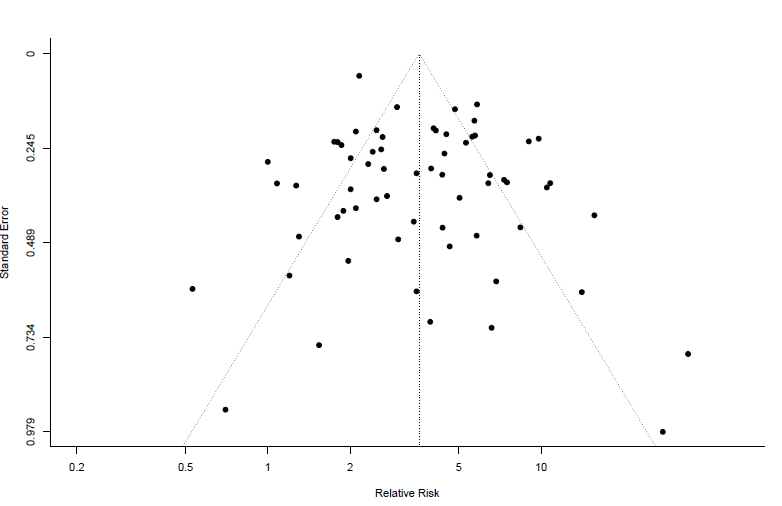


**A**


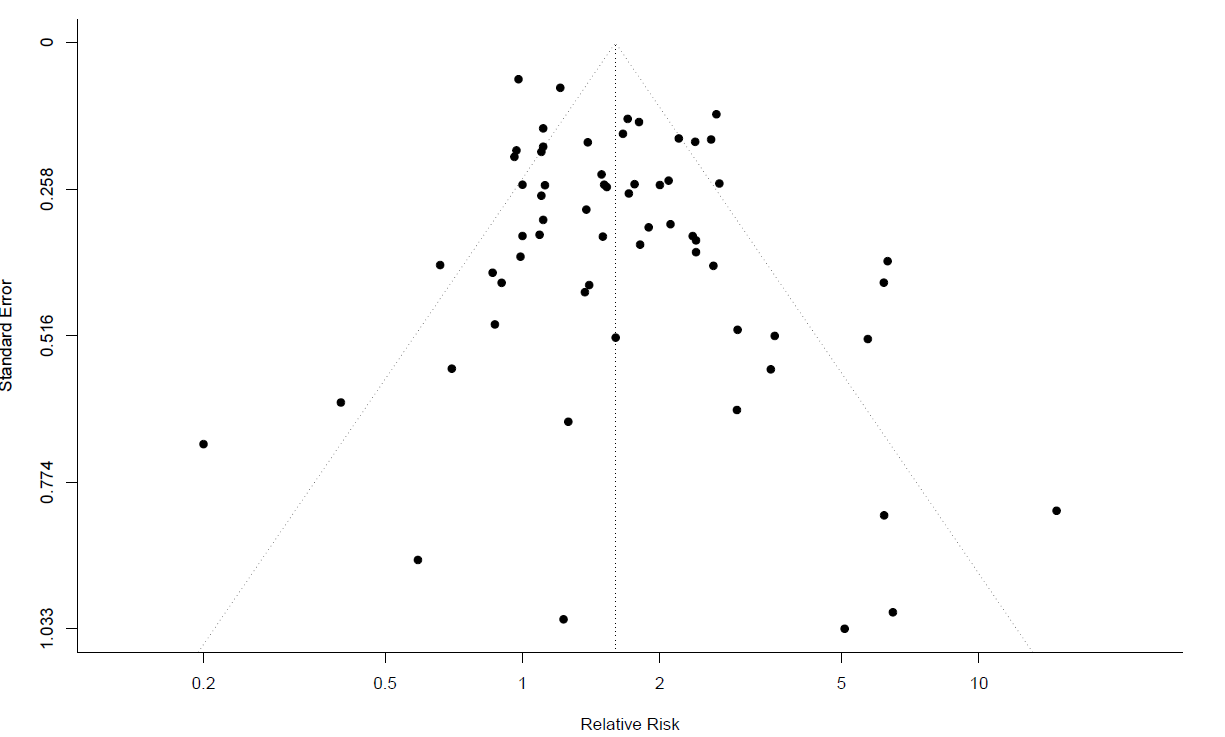


**B**


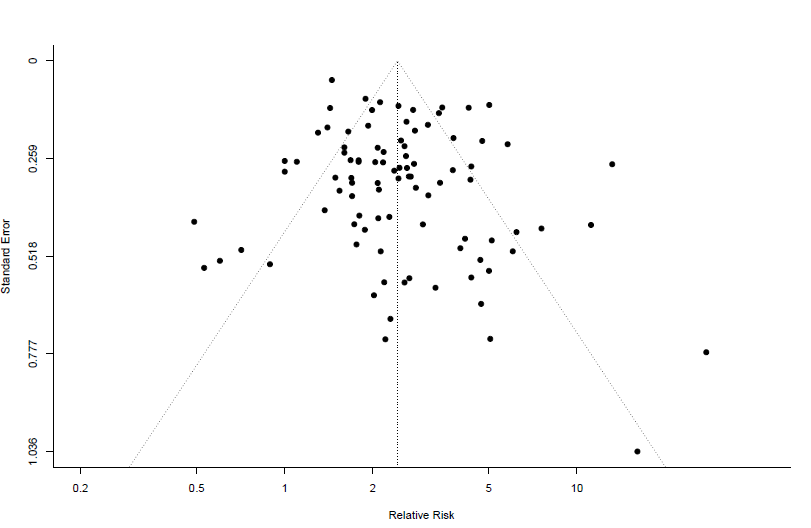


**C**

**Supplementary Table 8.** Comparison of original and trim-and-fill adjusted pooled relative risk (RR) and corresponding 95% confidence interval (CI) for oral and pharyngeal (OPC) risk for current, former, and ever cigarette smokers vs. never cigarette smokers.

|  | **Current Smokers**  **Pooled RR**  **(95% CI)** | **Former Smokers**  **Pooled RR**  **(95% CI)** | **Ever Smokers**  **Pooled RR**  **(95% CI)** |
| --- | --- | --- | --- |
| Original | 3.58  (3.03-4.24) | 1.61  (1.44-1.81) | 2.45  (2.19-2.75) |
| Trim-and-filled | 3.00  (2.48-3.63) | 1.44  (1.25-1.66) | 1.98  (1.73-2.27) |

**Supplementary Box 1.** Literature search strings for the update of the last available comprehensive review on the association between smoking and oral and pharyngeal cancer risk used in MEDLINE and Embase.

| **Source** | **Date** | **Search string** | **N** |
| --- | --- | --- | --- |
| PubMed | 05/02/2025 | (pharynx [tiab] OR pharyngeal [tiab] OR oral cavity [tiab]) AND (cancer OR neoplasm OR carcinoma OR adenocarcinoma OR Neoplasms [MeSH Terms]) AND (cigarette OR cigarettes OR tobacco OR smoking OR smokers OR smoking [MeSH Terms]) AND (English[Language]) AND ("2008"[Date - Publication] : "2025"[Date - Publication]) | 1779 |
| Embase | 05/02/2025 | cigarette:ti OR cigarettes:ti OR tobacco:ti OR smoking:ti OR smokers:ti AND (pharynx:ab,ti OR pharyngeal:ab,ti OR oral cavity:ab,ti) AND (cancer:ab,ti OR neoplasm:ab,ti OR carcinoma:ab,ti OR adenocarcinoma:ab,ti) AND (article:it OR review:it) AND [english]/lim AND [2008-2025]/py | 273 |
|  |  | Duplicates | 241 |
| **TOT** | 05/02/2025 | **-** | **1811 non duplicates** |

**Supplementary Box 2.** Functions of the splines and linear models used to estimate the associations between smoking intensity (current vs. never smokers), duration (current vs. never smokers) and time since quitting (former vs. current smokers) and the risk of oral and pharyngeal cancer.

| **Smoking intensity among current smokers (cigarettes/day)** |
| --- |
| $f\left( x \right)=\left\{ \begin{matrix} 0.117997 x - 0.000121424 x^{3} 0\leq x<10 \\ 0.0000578211 x^{3}- 0.00537736 x^{2}+ 0.171771 x - 0.179245 10\leq x<31 \\ 0.00507255 x + 1.5433 x\geq31 \end{matrix} \right.$ |
| **Smoking duration among current smokers (years)** |
| $f\left( x \right)=\left\{ \begin{matrix} 0.102482 x - 0.000051055 x^3 0\leq x<18 \\ 0.0000306093 x^3 - 0.00440774 x^2 + 0.181821 x - 0.476035 18\leq x<48 \\ 2.90911 - 0.0297503 x x\geq48 \end{matrix} \right.$ |
| **Time since quitting (years)** |
| $f\left( x \right)=-0.0724x$ |

**REFERENCES**

1. Ansary-Moghaddam A, Huxley RR, Lam TH, Woodward M. The risk of upper aero digestive tract cancer associated with smoking, with and without concurrent alcohol consumption. Mt Sinai J Med 2009; 76: 392-403.

2. Ansary-Moghaddam A, Martiniuk A, Lam TH et al. Smoking and the risk of upper aero digestive tract cancers for men and women in the Asia-Pacific region. Int J Environ Res Public Health 2009; 6: 1358-1370.

3. Asombang AW, Chishinga N, Nkhoma A et al. Systematic review and meta-analysis of esophageal cancer in Africa: Epidemiology, risk factors, management and outcomes. World J Gastroenterol 2019; 25: 4512-4533.

4. Bakkalci D, Jia Y, Winter JR et al. Risk factors for Epstein Barr virus-associated cancers: a systematic review, critical appraisal, and mapping of the epidemiological evidence. J Glob Health 2020; 10: 010405.

5. Berthiller J, Straif K, Agudo A et al. Low frequency of cigarette smoking and the risk of head and neck cancer in the INHANCE consortium pooled analysis. Int J Epidemiol 2016; 45: 835-845.

6. Carter BD, Abnet CC, Feskanich D et al. Smoking and mortality--beyond established causes. N Engl J Med 2015; 372: 631-640.

7. Castellsagué X, Muñoz N, De Stefani E et al. Independent and joint effects of tobacco smoking and alcohol drinking on the risk of esophageal cancer in men and women. Int J Cancer 1999; 82: 657-664.

8. Castellsagué X, Muñoz N, De Stefani E et al. Smoking and drinking cessation and risk of esophageal cancer (Spain). Cancer Causes Control 2000; 11: 813-818.

9. Castro C, Peleteiro B, Lunet N. Modifiable factors and esophageal cancer: a systematic review of published meta-analyses. J Gastroenterol 2018; 53: 37-51.

10. Chang CP, Chang SC, Chuang SC et al. Age at start of using tobacco on the risk of head and neck cancer: Pooled analysis in the International Head and Neck Cancer Epidemiology Consortium (INHANCE). Cancer Epidemiol 2019; 63: 101615.

11. Chetwood JD, Garg P, Finch P, Gordon M. Systematic review: the etiology of esophageal squamous cell carcinoma in low-income settings. Expert Rev Gastroenterol Hepatol 2019; 13: 71-88.

12. Cook MB, Kamangar F, Whiteman DC et al. Cigarette smoking and adenocarcinomas of the esophagus and esophagogastric junction: a pooled analysis from the international BEACON consortium. J Natl Cancer Inst 2010; 102: 1344-1353.

13. Di Credico G, Edefonti V, Polesel J et al. Joint effects of intensity and duration of cigarette smoking on the risk of head and neck cancer: A bivariate spline model approach. Oral Oncology 2019; 94: 47-57.

14. Drahos J, Xiao Q, Risch HA et al. Age-specific risk factor profiles of adenocarcinomas of the esophagus: A pooled analysis from the international BEACON consortium. Int J Cancer 2016; 138: 55-64.

15. Du L, Lei L, Zhao X et al. The Interaction of Smoking with Gene Polymorphisms on Four Digestive Cancers: A Systematic Review and Meta-Analysis. J Cancer 2018; 9: 1506-1517.

16. Fahey PP, Mallitt KA, Astell-Burt T et al. Impact of pre-diagnosis behavior on risk of death from esophageal cancer: a systematic review and meta-analysis. Cancer Causes Control 2015; 26: 1365-1373.

17. Gandini S, Botteri E, Iodice S et al. Tobacco smoking and cancer: a meta-analysis. Int J Cancer 2008; 122: 155-164.

18. Hashibe M, Brennan P, Benhamou S et al. Alcohol drinking in never users of tobacco, cigarette smoking in never drinkers, and the risk of head and neck cancer: pooled analysis in the International Head and Neck Cancer Epidemiology Consortium. J Natl Cancer Inst 2007; 99: 777-789.

19. Hashibe M, Brennan P, Chuang SC et al. Interaction between tobacco and alcohol use and the risk of head and neck cancer: pooled analysis in the International Head and Neck Cancer Epidemiology Consortium. Cancer Epidemiol Biomarkers Prev 2009; 18: 541-550.

20. IARC. Tobacco smoke and involuntary smoking. Vol. 83. IARC Monogr Eval Carcinog Risks Hum 2004; 1-1438.

21. IARC. Personal habits and indoor combustions. Volume 100 E. IARC Monogr Eval Carcinog Risks Hum 2012; 1-538.

22. Ishikawa A, Kuriyama S, Tsubono Y et al. Smoking, alcohol drinking, green tea consumption and the risk of esophageal cancer in Japanese men. J Epidemiol 2006; 16: 185-192.

23. Jia WH, Qin HD. Non-viral environmental risk factors for nasopharyngeal carcinoma: a systematic review. Semin Cancer Biol 2012; 22: 117-126.

24. Jones MR, Tellez-Plaza M, Navas-Acien A. Smoking, menthol cigarettes and all-cause, cancer and cardiovascular mortality: evidence from the National Health and Nutrition Examination Survey (NHANES) and a meta-analysis. PLoS One 2013; 8: e77941.

25. Khalifeh M, Ginex P, Boffetta P. Reduction of head and neck cancer risk following smoking cessation: a systematic review and meta-analysis. BMJ Open 2024; 14: e074723.

26. Katanoda K, Marugame T, Saika K et al. Population attributable fraction of mortality associated with tobacco smoking in Japan: a pooled analysis of three large-scale cohort studies. J Epidemiol 2008; 18: 251-264.

27. Khani Y, Pourgholam-Amiji N, Afshar M et al. Tobacco smoking and cancer types: A review. Biomedical Research and Therapy 2018; 5: 2142-2159.

28. Koyanagi YN, Matsuo K, Ito H et al. Cigarette smoking and the risk of head and neck cancer in the Japanese population: a systematic review and meta-analysis. Jpn J Clin Oncol 2016; 46: 580-595.

29. Lin JH, Wen CP, Jiang CQ et al. Smoking and nasopharyngeal cancer: individual data meta-analysis of six prospective studies on 334 935 men. Int J Epidemiol 2021; 50: 975-986.

30. Long M, Fu Z, Li P, Nie Z. Cigarette smoking and the risk of nasopharyngeal carcinoma: a meta-analysis of epidemiological studies. BMJ Open 2017; 7: e016582.

31. Lubin JH, Purdue M, Kelsey K et al. Total exposure and exposure rate effects for alcohol and smoking and risk of head and neck cancer: a pooled analysis of case-control studies. Am J Epidemiol 2009; 170: 937-947.

32. Lubin JH, Cook MB, Pandeya N et al. The importance of exposure rate on odds ratios by cigarette smoking and alcohol consumption for esophageal adenocarcinoma and squamous cell carcinoma in the Barrett's Esophagus and Esophageal Adenocarcinoma Consortium. Cancer Epidemiol 2012; 36: 306-316.

33. Macfarlane GJ, Zheng T, Marshall JR et al. Alcohol, tobacco, diet and the risk of oral cancer: a pooled analysis of three case-control studies. Eur J Cancer B Oral Oncol 1995; 31B: 181-187.

34. Mello FW, Melo G, Pasetto JJ et al. The synergistic effect of tobacco and alcohol consumption on oral squamous cell carcinoma: a systematic review and meta-analysis. Clin Oral Investig 2019; 23: 2849-2859.

35. Miyazaki T, Kitagawa Y, Kuwano H et al. Decreased risk of esophageal cancer owing to cigarette and alcohol cessation in smokers and drinkers: a systematic review and meta-analysis. Esophagus 2017; 14: 290-302.

36. Nakamura K, Huxley R, Ansary-Moghaddam A, Woodward M. The hazards and benefits associated with smoking and smoking cessation in Asia: a meta-analysis of prospective studies. Tob Control 2009; 18: 345-353.

37. Okekpa SI, RB SMNM, Mangantig E et al. Nasopharyngeal Carcinoma (NPC) Risk Factors: A Systematic Review and Meta-Analysis of the Association with Lifestyle, Diets, Socioeconomic and Sociodemographic in Asian Region. Asian Pac J Cancer Prev 2019; 20: 3505-3514.

38. Ordóñez-Mena JM, Schöttker B, Mons U et al. Quantification of the smoking-associated cancer risk with rate advancement periods: meta-analysis of individual participant data from cohorts of the CHANCES consortium. BMC Med 2016; 14: 62.

39. Oze I, Matsuo K, Ito H et al. Cigarette smoking and esophageal cancer risk: an evaluation based on a systematic review of epidemiologic evidence among the Japanese population. Jpn J Clin Oncol 2012; 42: 63-73.

40. Oze I, Charvat H, Matsuo K et al. Revisit of an unanswered question by pooled analysis of eight cohort studies in Japan: Does cigarette smoking and alcohol drinking have interaction for the risk of esophageal cancer? Cancer Med 2019; 8: 6414-6425.

41. Romdhoni AC, Rejeki PS, Guo HR et al. Risk Factors Associated with Nasopharyngeal Cancer Incidences in Indonesia: A Systematic Review and Meta-Analysis. Asian Pac J Cancer Prev 2023; 24: 1105-1111.

42. Park S, Jee SH, Shin HR et al. Attributable fraction of tobacco smoking on cancer using population-based nationwide cancer incidence and mortality data in Korea. BMC Cancer 2014; 14: 406.

43. Petti S, Masood M, Scully C. The magnitude of tobacco smoking-betel quid chewing-alcohol drinking interaction effect on oral cancer in South-East Asia. A meta-analysis of observational studies. PLoS One 2013; 8: e78999.

44. Prabhu A, Obi KO, Rubenstein JH. Systematic review with meta-analysis: race-specific effects of alcohol and tobacco on the risk of oesophageal squamous cell carcinoma. Aliment Pharmacol Ther 2013; 38: 1145-1155.

45. Prabhu A, Obi KO, Rubenstein JH. The synergistic effects of alcohol and tobacco consumption on the risk of esophageal squamous cell carcinoma: a meta-analysis. Am J Gastroenterol 2014; 109: 822-827.

46. Prasad JB, Dhar M. Risk of major cancers associated with various forms of tobacco use in India: a systematic review and meta-analysis. Journal of Public Health (Germany) 2019; 27: 803-813.

47. Sadri G, Mahjub H. Tobacco smoking and oral cancer: a meta-analysis. J Res Health Sci 2007; 7: 18-23.

48. Saito E, Inoue M, Tsugane S et al. Smoking cessation and subsequent risk of cancer: A pooled analysis of eight population-based cohort studies in Japan. Cancer Epidemiol 2017; 51: 98-108.

49. t Mannetje A, Kogevinas M, Luce D et al. Sinonasal cancer, occupation, and tobacco smoking in European women and men. Am J Ind Med 1999; 36: 101-107.

50. Toporcov TN, Znaor A, Zhang ZF et al. Risk factors for head and neck cancer in young adults: a pooled analysis in the INHANCE consortium. Int J Epidemiol 2015; 44: 169-185.

51. Tramacere I, La Vecchia C, Negri E. Tobacco smoking and esophageal and gastric cardia adenocarcinoma: a meta-analysis. Epidemiology 2011; 22: 344-349.

52. U.S. Department of Health and Human Services. Women and Smoking: A Report of the Surgeon General. 2001.

53. U.S. Department of Health and Human Services. The Health Consequences of Smoking: A Report of the Surgeon General. Atlanta, GA: Centers for Disease Control and Prevention, National Center for Chronic Disease Prevention and Health Promotion, Office on Smoking and Health,2004.

54. Wang QL, Xie SH, Li WT, Lagergren J. Smoking Cessation and Risk of Esophageal Cancer by Histological Type: Systematic Review and Meta-analysis. J Natl Cancer Inst 2017; 109.

55. Wyss A, Hashibe M, Chuang SC et al. Cigarette, cigar, and pipe smoking and the risk of head and neck cancers: pooled analysis in the International Head and Neck Cancer Epidemiology Consortium. Am J Epidemiol 2013; 178: 679-690.

56. Xue WQ, Qin HD, Ruan HL et al. Quantitative association of tobacco smoking with the risk of nasopharyngeal carcinoma: a comprehensive meta-analysis of studies conducted between 1979 and 2011. Am J Epidemiol 2013; 178: 325-338.

57. Yu KT, Ge C, Xu XF et al. CYP1A1 polymorphism interactions with smoking status in oral cancer risk: evidence from epidemiological studies. Tumour Biol 2014; 35: 11183-11191.

58. Zeka A, Gore R, Kriebel D. Effects of alcohol and tobacco on aerodigestive cancer risks: a meta-regression analysis. Cancer Causes Control 2003; 14: 897-906.

59. Zhang ZJ, Hao K, Shi R et al. Glutathione S-transferase M1 (GSTM1) and glutathione S-transferase T1 (GSTT1) null polymorphisms, smoking, and their interaction in oral cancer: a HuGE review and meta-analysis. Am J Epidemiol 2011; 173: 847-857.

60. Zheng W, McLerran DF, Rolland BA et al. Burden of total and cause-specific mortality related to tobacco smoking among adults aged ≥ 45 years in Asia: a pooled analysis of 21 cohorts. PLoS Med 2014; 11: e1001631.

61. Zuo JJ, Tao ZZ, Chen C et al. Characteristics of cigarette smoking without alcohol consumption and laryngeal cancer: overall and time-risk relation. A meta-analysis of observational studies. Eur Arch Otorhinolaryngol 2017; 274: 1617-1631.

62. Allen NE, Beral V, Casabonne D et al. Moderate alcohol intake and cancer incidence in women. J Natl Cancer Inst 2009; 101: 296-305.

63. Anantharaman D, Chaubal PM, Kannan S et al. Susceptibility to oral cancer by genetic polymorphisms at CYP1A1, GSTM1 and GSTT1 loci among Indians: tobacco exposure as a risk modulator. Carcinogenesis 2007; 28: 1455-1462.

64. Andrade JO, Santos CA, Oliveira MC. Associated factors with oral cancer: a study of case control in a population of the Brazil's Northeast. Rev Bras Epidemiol 2015; 18: 894-905.

65. Andreotti M, Rodrigues AN, Cardoso LM et al. [Occupational status and cancer of the oral cavity and oropharynx]. Cad Saude Publica 2006; 22: 543-552.

66. Benhamou S, Tuimala J, Bouchardy C et al. DNA repair gene XRCC2 and XRCC3 polymorphisms and susceptibility to cancers of the upper aerodigestive tract. Int J Cancer 2004; 112: 901-904.

67. Boffetta P, Mashberg A, Winkelmann R, Garfinkel L. Carcinogenic effect of tobacco smoking and alcohol drinking on anatomic sites of the oral cavity and oropharynx. Int J Cancer 1992; 52: 530-533.

68. Bosetti C, Gallus S, Trichopoulou A et al. Influence of the Mediterranean diet on the risk of cancers of the upper aerodigestive tract. Cancer Epidemiol Biomarkers Prev 2003; 12: 1091-1094.

69. Bravi F, Bosetti C, Filomeno M et al. Foods, nutrients and the risk of oral and pharyngeal cancer. Br J Cancer 2013; 109: 2904-2910.

70. Bross ID, Coombs J. Early onset of oral cancer among women who drink and smoke. Oncology 1976; 33: 136-139.

71. Brugere J, Guenel P, Leclerc A, Rodriguez J. Differential effects of tobacco and alcohol in cancer of the larynx, pharynx, and mouth. Cancer 1986; 57: 391-395.

72. Buch SC, Notani PN, Bhisey RA. Polymorphism at GSTM1, GSTM3 and GSTT1 gene loci and susceptibility to oral cancer in an Indian population. Carcinogenesis 2002; 23: 803-807.

73. Canova C, Richiardi L, Merletti F et al. Alcohol, tobacco and genetic susceptibility in relation to cancers of the upper aerodigestive tract in northern Italy. Tumori 2010; 96: 1-10.

74. Cha IH, Park JY, Chung WY et al. Polymorphisms of CYP1A1 and GSTM1 genes and susceptibility to oral cancer. Yonsei Med J 2007; 48: 233-239.

75. Chang IH, Jiang RS, Wong YK et al. Visual screening of oral cavity cancer in a male population: experience from a medical center. J Chin Med Assoc 2011; 74: 561-566.

76. Chatterjee S, Dhar S, Sengupta B et al. Polymorphisms of CYP1A1, GSTM1 and GSTT1 Loci as the Genetic Predispositions of Oral Cancers and Other Oral Pathologies: Tobacco and Alcohol as Risk Modifiers. Indian J Clin Biochem 2010; 25: 260-272.

77. Choi S, Kahyo H, Shim Y. Effect of cigarette smoking and alcohol drinking on risk of cancers. Korean J Epidemiol 1992; 14: 35-53.

78. Chyou PH, Nomura AM, Stemmermann GN. Diet, alcohol, smoking and cancer of the upper aerodigestive tract: a prospective study among Hawaii Japanese men. Int J Cancer 1995; 60: 616-621.

79. Cordero K, Espinoza I, Caceres D et al. Oral cancer susceptibility associated with the CYP1A1 and GSTM1 genotypes in Chilean individuals. Oncol Lett 2010; 1: 549-553.

80. Cui Y, Morgenstern H, Greenland S et al. Polymorphism of Xeroderma Pigmentosum group G and the risk of lung cancer and squamous cell carcinomas of the oropharynx, larynx and esophagus. Int J Cancer 2006; 118: 714-720.

81. De Stefani E, Oreggia F, Rivero S, Fierro L. Hand-rolled cigarette smoking and risk of cancer of the mouth, pharynx, and larynx. Cancer 1992; 70: 679-682.

82. Divaris K, Olshan AF, Smith J et al. Oral health and risk for head and neck squamous cell carcinoma: the Carolina Head and Neck Cancer Study. Cancer Causes Control 2010; 21: 567-575.

83. Doll R, Peto R, Boreham J, Sutherland I. Mortality from cancer in relation to smoking: 50 years observations on British doctors. Br J Cancer 2005; 92: 426-429.

84. Elwood JM, Pearson JC, Skippen DH, Jackson SM. Alcohol, smoking, social and occupational factors in the aetiology of cancer of the oral cavity, pharynx and larynx. Int J Cancer 1984; 34: 603-612.

85. Galli P, Cadoni G, Volante M et al. A case-control study on the combined effects of p53 and p73 polymorphisms on head and neck cancer risk in an Italian population. BMC Cancer 2009; 9: 137.

86. Gronau S, Koenig-Greger D, Jerg M, Riechelmann H. GSTM1 enzyme concentration and enzyme activity in correlation to the genotype of detoxification enzymes in squamous cell carcinoma of the oral cavity. Oral Dis 2003; 9: 62-67.

87. Guha N, Boffetta P, Wunsch Filho V et al. Oral health and risk of squamous cell carcinoma of the head and neck and esophagus: results of two multicentric case-control studies. Am J Epidemiol 2007; 166: 1159-1173.

88. Guo L, Zhang C, Shi S, Guo X. [Correlation between smoking and the polymorphisms of cytochrome P450 1A1-Msp I and glutathione S-transferase T1 genes and oral cancer]. Hua Xi Kou Qiang Yi Xue Za Zhi 2012; 30: 187-191.

89. Hammond EC, Horn D. Smoking and death rates: report on forty-four months of follow-up of 187,783 men. 2. Death rates by cause. J Am Med Assoc 1958; 166: 1294-1308.

90. Hashibe M, Boffetta P, Zaridze D et al. Evidence for an important role of alcohol- and aldehyde-metabolizing genes in cancers of the upper aerodigestive tract. Cancer Epidemiol Biomarkers Prev 2006; 15: 696-703.

91. Hashibe M, Brennan P, Benhamou S et al. Alcohol drinking in never users of tobacco, cigarette smoking in never drinkers, and the risk of head and neck cancer: pooled analysis in the International Head and Neck Cancer Epidemiology Consortium. J Natl Cancer Inst 2007; 99: 777-789.

92. Henderson BE, Louie E, SooHoo Jing J et al. Risk factors associated with nasopharyngeal carcinoma. N Engl J Med 1976; 295: 1101-1106.

93. Hirayama T. Life-style and mortality. A large-scale census-based cohort study in Japan. Basel, Switzerland: Karger 1990.

94. Idris AM, Nair J, Ohshima H et al. Unusually high levels of carcinogenic tobacco-specific nitrosamines in Sudan snuff (toombak). Carcinogenesis 1991; 12: 1115-1118.

95. Jaber MA, Porter SR, Gilthorpe MS et al. Risk factors for oral epithelial dysplasia--the role of smoking and alcohol. Oral Oncol 1999; 35: 151-156.

96. Jayaprakash V, Rigual NR, Moysich KB et al. Chemoprevention of head and neck cancer with aspirin: a case-control study. Arch Otolaryngol Head Neck Surg 2006; 132: 1231-1236.

97. Kabat GC, Hebert JR. Use of mentholated cigarettes and oropharyngeal cancer. Epidemiology 1994; 5: 183-188.

98. Kamiyama I, Shono T, Narita M. A study of drinking and smoking as carcinogens in the oral cancer. Shikwa Gakuho 2005; 105:446–152.

99. Kao SY, Wu CH, Lin SC et al. Genetic polymorphism of cytochrome P4501A1 and susceptibility to oral squamous cell carcinoma and oral precancer lesions associated with smoking/betel use. J Oral Pathol Med 2002; 31: 505-511.

100. Keller AZ, Terris M. The association of alcohol and tobacco with cancer of the mouth and pharynx. Am J Public Health Nations Health 1965; 55: 1578-1585.

101. La Vecchia C, Negri E, D'Avanzo B et al. Dietary indicators of oral and pharyngeal cancer. Int J Epidemiol 1991; 20: 39-44.

102. La Vecchia C, Talamini R, Bosetti C et al. RESPONSE: re: cancer of the oral cavity and pharynx in nonsmokers who drink alcohol and in nondrinkers who smoke tobacco. J Natl Cancer Inst 1999; 91: 1337-1338.

103. Lee KD, Lu CH, Chen PT et al. The incidence and risk of developing a second primary esophageal cancer in patients with oral and pharyngeal carcinoma: a population-based study in Taiwan over a 25 year period. BMC Cancer 2009; 9: 373.

104. Lee KD, Wang TY, Lu CH et al. The bidirectional association between oral cancer and esophageal cancer: A population-based study in Taiwan over a 28-year period. Oncotarget 2017; 8: 44567-44578.

105. Levi F, Pasche C, La Vecchia C et al. Food groups and risk of oral and pharyngeal cancer. Int J Cancer 1998; 77: 705-709.

106. Lin WJ, Jiang RS, Wu SH et al. Smoking, alcohol, and betel quid and oral cancer: a prospective cohort study. J Oncol 2011; 2011: 525976.

107. Lohe VK, Degwekar SS, Bhowate RR et al. Evaluation of correlation of serum lipid profile in patients with oral cancer and precancer and its association with tobacco abuse. J Oral Pathol Med 2010; 39: 141-148.

108. Losi-Guembarovski R, Colus IM, De Menezes RP et al. Lack of association among polymorphic xenobiotic-metabolizing enzyme genotypes and the occurrence and progression of oral carcinoma in a Brazilian population. Anticancer Res 2008; 28: 1023-1028.

109. Maier H, Dietz A, Gewelke U et al. Tobacco and alcohol and the risk of head and neck cancer. Clin Investig 1992; 70: 320-327.

110. Maier H, Sennewald E, Heller GF, Weidauer H. Chronic alcohol consumption--the key risk factor for pharyngeal cancer. Otolaryngol Head Neck Surg 1994; 110: 168-173.

111. Mashberg A, Boffetta P, Winkelman R, Garfinkel L. Tobacco smoking, alcohol drinking, and cancer of the oral cavity and oropharynx among U.S. veterans. Cancer 1993; 72: 1369-1375.

112. Menvielle G, Luce D, Goldberg P et al. Smoking, alcohol drinking and cancer risk for various sites of the larynx and hypopharynx. A case-control study in France. Eur J Cancer Prev 2004; 13: 165-172.

113. Moura MA, Bergmann A, Aguiar SS, Thuler LC. The magnitude of the association between smoking and the risk of developing cancer in Brazil: a multicenter study. BMJ Open 2014; 4: e003736.

114. Nasher AT, Al-Hebshi NN, Al-Moayad EE, Suleiman AM. Viral infection and oral habits as risk factors for oral squamous cell carcinoma in Yemen: a case-control study. Oral Surg Oral Med Oral Pathol Oral Radiol 2014; 118: 566-572 e561.

115. Notani PN. Role of alcohol in cancers of the upper alimentary tract: use of models in risk assessment. J Epidemiol Community Health 1988; 42: 187-192.

116. Oreggia F, De Stefani E, Correa P, Fierro L. Risk factors for cancer of the tongue in Uruguay. Cancer 1991; 67: 180-183.

117. Pacella-Norman R, Urban MI, Sitas F et al. Risk factors for oesophageal, lung, oral and laryngeal cancers in black South Africans. Br J Cancer 2002; 86: 1751-1756.

118. Peters ES, McClean MD, Marsit CJ et al. Glutathione S-transferase polymorphisms and the synergy of alcohol and tobacco in oral, pharyngeal, and laryngeal carcinoma. Cancer Epidemiol Biomarkers Prev 2006; 15: 2196-2202.

119. Preston-Martin S, Thomas DC, White SC, Cohen D. Prior exposure to medical and dental x-rays related to tumors of the parotid gland. J Natl Cancer Inst 1988; 80: 943-949.

120. Randi G, Scotti L, Bosetti C et al. Pipe smoking and cancers of the upper digestive tract. Int J Cancer 2007; 121: 2049-2051.

121. Rao DN, Ganesh B, Rao RS, Desai PB. Risk assessment of tobacco, alcohol and diet in oral cancer--a case-control study. Int J Cancer 1994; 58: 469-473.

122. Ray JG, Ganguly M, Rao BS et al. Clinico-epidemiological profile of oral potentially malignant and malignant conditions among areca nut, tobacco and alcohol users in Eastern India: A hospital based study. J Oral Maxillofac Pathol 2013; 17: 45-50.

123. Rogers MA, Thomas DB, Davis S et al. A case-control study of element levels and cancer of the upper aerodigestive tract. Cancer Epidemiol Biomarkers Prev 1993; 2: 305-312.

124. Rosenblatt KA, Daling JR, Chen C et al. Marijuana use and risk of oral squamous cell carcinoma. Cancer Res 2004; 64: 4049-4054.

125. Rosenquist K. Risk factors in oral and oropharyngeal squamous cell carcinoma: a population-based case-control study in southern Sweden. Swed Dent J Suppl 2005; 1-66.

126. Sanderson RJ, de Boer MF, Damhuis RA et al. The influence of alcohol and smoking on the incidence of oral and oropharyngeal cancer in women. Clin Otolaryngol Allied Sci 1997; 22: 444-448.

127. Sankaranarayanan R, Duffy SW, Padmakumary G et al. Tobacco chewing, alcohol and nasal snuff in cancer of the gingiva in Kerala, India. Br J Cancer 1989; 60: 638-643.

128. Sankaranarayanan R, Duffy SW, Padmakumary G et al. Risk factors for cancer of the buccal and labial mucosa in Kerala, southern India. J Epidemiol Community Health 1990; 44: 286-292.

129. Sato M, Sato T, Izumo T, Amagasa T. Genetic polymorphism of drug-metabolizing enzymes and susceptibility to oral cancer. Carcinogenesis 1999; 20: 1927-1931.

130. Sato F, Oze I, Kawakita D et al. Inverse association between toothbrushing and upper aerodigestive tract cancer risk in a Japanese population. Head Neck 2011; 33: 1628-1637.

131. Sharma A, Mishra A, Das BC et al. Genetic polymorphism at GSTM1 and GSTT1 gene loci and susceptibility to oral cancer. Neoplasma 2006; 53: 309-315.

132. Shiu MN, Chen TH. Impact of betel quid, tobacco and alcohol on three-stage disease natural history of oral leukoplakia and cancer: implication for prevention of oral cancer. Eur J Cancer Prev 2004; 13: 39-45.

133. Shukla D, Dinesh Kale A, Hallikerimath S et al. Genetic polymorphism of drug metabolizing enzymes (GSTM1 and CYP1A1) as risk factors for oral premalignant lesions and oral cancer. Biomed Pap Med Fac Univ Palacky Olomouc Czech Repub 2012; 156: 253-259.

134. Singh RD, Haridas N, Shah FD et al. Gene polymorphisms, tobacco exposure and oral cancer susceptibility: a study from Gujarat, West India. Oral Dis 2014; 20: 84-93.

135. Smith EM, Ritchie JM, Summersgill KF et al. Age, sexual behavior and human papillomavirus infection in oral cavity and oropharyngeal cancers. Int J Cancer 2004; 108: 766-772.

136. Sreelekha TT, Ramadas K, Pandey M et al. Genetic polymorphism of CYP1A1, GSTM1 and GSTT1 genes in Indian oral cancer. Oral Oncol 2001; 37: 593-598.

137. Suzuki T, Wakai K, Matsuo K et al. Effect of dietary antioxidants and risk of oral, pharyngeal and laryngeal squamous cell carcinoma according to smoking and drinking habits. Cancer Sci 2006; 97: 760-767.

138. Talamini R, Franceschi S, Barra S, La Vecchia C. The role of alcohol in oral and pharyngeal cancer in non-smokers, and of tobacco in non-drinkers. Int J Cancer 1990; 46: 391-393.

139. Talamini R, La Vecchia C, Levi F et al. Cancer of the oral cavity and pharynx in nonsmokers who drink alcohol and in nondrinkers who smoke tobacco. J Natl Cancer Inst 1998; 90: 1901-1903.

140. Thomas SJ, Bain CJ, Battistutta D et al. Betel quid not containing tobacco and oral cancer: a report on a case-control study in Papua New Guinea and a meta-analysis of current evidence. Int J Cancer 2007; 120: 1318-1323.

141. Tsai KY, Su CC, Lin YY et al. Quantification of betel quid chewing and cigarette smoking in oral cancer patients. Community Dent Oral Epidemiol 2009; 37: 555-561.

142. Vincent RG, Marchetta F. The Relationship of the Use of Tobacco and Alcohol to Cancer of the Oral Cavity, Pharynx or Larynx. Am J Surg 1963; 106: 501-505.

143. Weikert C, Dietrich T, Boeing H et al. Lifetime and baseline alcohol intake and risk of cancer of the upper aero-digestive tract in the European Prospective Investigation into Cancer and Nutrition (EPIC) study. Int J Cancer 2009; 125: 406-412.

144. Weir JM, Dunn JE, Jr. Smoking and mortality: a prospective study. Cancer 1970; 25: 105-112.

145. Williams RR, Horm JW. Association of cancer sites with tobacco and alcohol consumption and socioeconomic status of patients: interview study from the Third National Cancer Survey. J Natl Cancer Inst 1977; 58: 525-547.

146. Wynder EL, Stellman SD. Comparative epidemiology of tobacco-related cancers. Cancer Res 1977; 37: 4608-4622.

147. Yang KY, Jiang RS, Shiao JY et al. Visual screening of oral cavity cancer: The role of otolaryngologists. Laryngoscope 2007; 117: 92-95.

148. Yen TT, Lin WD, Wang CP et al. The association of smoking, alcoholic consumption, betel quid chewing and oral cavity cancer: a cohort study. Eur Arch Otorhinolaryngol 2008; 265: 1403-1407.

149. Zhang Z, Shi Q, Liu Z et al. Polymorphisms of methionine synthase and methionine synthase reductase and risk of squamous cell carcinoma of the head and neck: a case-control analysis. Cancer Epidemiol Biomarkers Prev 2005; 14: 1188-1193.

150. Zheng TZ, Boyle P, Hu HF et al. Dentition, oral hygiene, and risk of oral cancer: a case-control study in Beijing, People's Republic of China. Cancer Causes Control 1990; 1: 235-241.

151. Zheng W, Blot WJ, Shu XO et al. Risk factors for oral and pharyngeal cancer in Shanghai, with emphasis on diet. Cancer Epidemiol Biomarkers Prev 1992; 1: 441-448.

152. Anantharaman D, Marron M, Lagiou P et al. Population attributable risk of tobacco and alcohol for upper aerodigestive tract cancer. Oral Oncol 2011; 47: 725-731.

153. Lee YC, Marron M, Benhamou S et al. Active and involuntary tobacco smoking and upper aerodigestive tract cancer risks in a multicenter case-control study. Cancer Epidemiol Biomarkers Prev 2009; 18: 3353-3361.

154. Baron AE, Franceschi S, Barra S et al. A comparison of the joint effects of alcohol and smoking on the risk of cancer across sites in the upper aerodigestive tract. Cancer Epidemiol Biomarkers Prev 1993; 2: 519-523.

155. La Vecchia C, Franceschi S, Bosetti C et al. Time since stopping smoking and the risk of oral and pharyngeal cancers. J Natl Cancer Inst 1999; 91: 726-728.

156. Barra S, Baron AE, Franceschi S et al. Cancer and non-cancer controls in studies on the effect of tobacco and alcohol consumption. Int J Epidemiol 1991; 20: 845-851.

157. Bosetti C, Gallus S, Peto R et al. Tobacco smoking, smoking cessation, and cumulative risk of upper aerodigestive tract cancers. Am J Epidemiol 2008; 167: 468-473.

158. Bosetti C, Negri E, Franceschi S et al. Risk factors for oral and pharyngeal cancer in women: a study from Italy and Switzerland. Br J Cancer 2000; 82: 204-207.

159. De Stefani E, Boffetta P, Oreggia F et al. Smoking patterns and cancer of the oral cavity and pharynx: a case-control study in Uruguay. Oral Oncol 1998; 34: 340-346.

160. De Stefani E, Boffetta P, Deneo-Pellegrini H et al. The effect of smoking and drinking in oral and pharyngeal cancers: a case-control study in Uruguay. Cancer Lett 2007; 246: 282-289.

161. Ferraroni M, Negri E, La Vecchia C et al. Socioeconomic indicators, tobacco and alcohol in the aetiology of digestive tract neoplasms. Int J Epidemiol 1989; 18: 556-562.

162. Franceschi S, Barra S, La Vecchia C et al. Risk factors for cancer of the tongue and the mouth. A case-control study from northern Italy. Cancer 1992; 70: 2227-2233.

163. Franceschi S, Levi F, La Vecchia C et al. Comparison of the effect of smoking and alcohol drinking between oral and pharyngeal cancer. Int J Cancer 1999; 83: 1-4.

164. Gallus S, Altieri A, Bosetti C et al. Cigarette tar yield and risk of upper digestive tract cancers: case-control studies from Italy and Switzerland. Ann Oncol 2003; 14: 209-213.

165. Galeone C, Edefonti V, Parpinel M et al. Folate intake and the risk of oral cavity and pharyngeal cancer: a pooled analysis within the International Head and Neck Cancer Epidemiology Consortium. Int J Cancer 2015; 136: 904-914.

166. Herrero R, Castellsague X, Pawlita M et al. Human papillomavirus and oral cancer: the International Agency for Research on Cancer multicenter study. J Natl Cancer Inst 2003; 95: 1772-1783.

167. Huang SJ, Tseng YK, Lo YH et al. Association of SDF-1 and CXCR4 Polymorphisms With Susceptibility to Oral and Pharyngeal Squamous Cell Carcinoma. Anticancer Res 2019; 39: 2891-2902.

168. Yang CM, Chen HC, Hou YY et al. A high IL-4 production diplotype is associated with an increased risk but better prognosis of oral and pharyngeal carcinomas. Arch Oral Biol 2014; 59: 35-46.

169. Inoue-Choi M, Shiels MS, McNeel TS et al. Contemporary Associations of Exclusive Cigarette, Cigar, Pipe, and Smokeless Tobacco Use With Overall and Cause-Specific Mortality in the United States. JNCI Cancer Spectr 2019; 3: pkz036.

170. Coleman NC, Burnett RT, Higbee JD et al. Cancer mortality risk, fine particulate air pollution, and smoking in a large, representative cohort of US adults. Cancer Causes Control 2020.

171. Lubin JH, Gaudet MM, Olshan AF et al. Body mass index, cigarette smoking, and alcohol consumption and cancers of the oral cavity, pharynx, and larynx: modeling odds ratios in pooled case-control data. Am J Epidemiol 2010; 171: 1250-1261.

172. Marron M, Boffetta P, Zhang ZF et al. Cessation of alcohol drinking, tobacco smoking and the reversal of head and neck cancer risk. Int J Epidemiol 2010; 39: 182-196.

173. Muscat JE, Richie JP, Jr., Thompson S, Wynder EL. Gender differences in smoking and risk for oral cancer. Cancer Res 1996; 56: 5192-5197.

174. Kabat GC, Chang CJ, Wynder EL. The role of tobacco, alcohol use, and body mass index in oral and pharyngeal cancer. Int J Epidemiol 1994; 23: 1137-1144.

175. Negri E, La Vecchia C, Franceschi S, Tavani A. Attributable risk for oral cancer in northern Italy. Cancer Epidemiol Biomarkers Prev 1993; 2: 189-193.

176. Peters ES, McClean MD, Liu M et al. The ADH1C polymorphism modifies the risk of squamous cell carcinoma of the head and neck associated with alcohol and tobacco use. Cancer Epidemiol Biomarkers Prev 2005; 14: 476-482.

177. Applebaum KM, Furniss CS, Zeka A et al. Lack of association of alcohol and tobacco with HPV16-associated head and neck cancer. J Natl Cancer Inst 2007; 99: 1801-1810.

178. Radoï L, Paget-Bailly S, Menvielle G et al. Tea and coffee consumption and risk of oral cavity cancer: results of a large population-based case-control study, the ICARE study. Cancer Epidemiol 2013; 37: 284-289.

179. Radoi L, Paget-Bailly S, Cyr D et al. Tobacco smoking, alcohol drinking and risk of oral cavity cancer by subsite: results of a French population-based case-control study, the ICARE study. Eur J Cancer Prev 2013; 22: 268-276.

180. Sanchez MJ, Martinez C, Nieto A et al. Oral and oropharyngeal cancer in Spain: influence of dietary patterns. Eur J Cancer Prev 2003; 12: 49-56.

181. Schlecht NF, Franco EL, Pintos J et al. Interaction between tobacco and alcohol consumption and the risk of cancers of the upper aero-digestive tract in Brazil. Am J Epidemiol 1999; 150: 1129-1137.

182. Schlecht NF, Franco EL, Pintos J, Kowalski LP. Effect of smoking cessation and tobacco type on the risk of cancers of the upper aero-digestive tract in Brazil. Epidemiology 1999; 10: 412-418.

183. Xie H, Hou L, Shields PG et al. Metabolic polymorphisms, smoking, and oral cancer in Puerto Rico. Oncol Res 2004; 14: 315-320.

184. Hayes RB, Bravo-Otero E, Kleinman DV et al. Tobacco and alcohol use and oral cancer in Puerto Rico. Cancer Causes Control 1999; 10: 27-33.

185. Zheng T, Holford T, Chen Y et al. Risk of tongue cancer associated with tobacco smoking and alcohol consumption: a case-control study. Oral Oncol 1997; 33: 82-85.

186. Zheng TZ, Boyle P, Hu HF et al. Tobacco smoking, alcohol consumption, and risk of oral cancer: a case-control study in Beijing, People's Republic of China. Cancer Causes Control 1990; 1: 173-179.

187. Adeyemi BF, Olusanya AA, Lawoyin JO. Oral squamous cell carcinoma, socioeconomic status and history of exposure to alcohol and tobacco. J Natl Med Assoc 2011; 103: 498-502.

188. Anantharaman D, Muller DC, Lagiou P et al. Combined effects of smoking and HPV16 in oropharyngeal cancer. Int J Epidemiol 2016; 45: 752-761.

189. Balaram P, Sridhar H, Rajkumar T et al. Oral cancer in southern India: the influence of smoking, drinking, paan-chewing and oral hygiene. Int J Cancer 2002; 98: 440-445.

190. Blot WJ, McLaughlin JK, Winn DM et al. Smoking and drinking in relation to oral and pharyngeal cancer. Cancer Res 1988; 48: 3282-3287.

191. Bundgaard T, Wildt J, Frydenberg M et al. Case-control study of squamous cell cancer of the oral cavity in Denmark. Cancer Causes Control 1995; 6: 57-67.

192. Castellsague X, Quintana MJ, Martinez MC et al. The role of type of tobacco and type of alcoholic beverage in oral carcinogenesis. Int J Cancer 2004; 108: 741-749.

193. Chandran R, Lalloo R, Myburgh NG, Chandran TM. Scientific. Risk of intraoral cancer associated with tobacco and alcohol--a case-control study. SADJ 2005; 60: 326-328.

194. Chen C, Ricks S, Doody DR et al. N-Acetyltransferase 2 polymorphisms, cigarette smoking and alcohol consumption, and oral squamous cell cancer risk. Carcinogenesis 2001; 22: 1993-1999.

195. Choi SY, Kahyo H. Effect of cigarette smoking and alcohol consumption in the aetiology of cancer of the oral cavity, pharynx and larynx. Int J Epidemiol 1991; 20: 878-885.

196. Day GL, Blot WJ, Austin DF et al. Racial differences in risk of oral and pharyngeal cancer: alcohol, tobacco, and other determinants. J Natl Cancer Inst 1993; 85: 465-473.

197. De Stefani E, Oreggia F, Ronco A et al. Salted meat consumption as a risk factor for cancer of the oral cavity and pharynx: a case-control study from Uruguay. Cancer Epidemiol Biomarkers Prev 1994; 3: 381-385.

198. Dikshit RP, Kanhere S. Tobacco habits and risk of lung, oropharyngeal and oral cavity cancer: a population-based case-control study in Bhopal, India. Int J Epidemiol 2000; 29: 609-614.

199. D'Souza G, Kreimer AR, Viscidi R et al. Case-control study of human papillomavirus and oropharyngeal cancer. N Engl J Med 2007; 356: 1944-1956.

200. Escribano Uzcudun A, Rabanal Retolaza I, Garcia Grande A et al. Pharyngeal cancer prevention: evidence from a case--control study involving 232 consecutive patients. J Laryngol Otol 2002; 116: 523-531.

201. Franceschi S, Talamini R, Barra S et al. Smoking and drinking in relation to cancers of the oral cavity, pharynx, larynx, and esophagus in northern Italy. Cancer Res 1990; 50: 6502-6507.

202. Garrote LF, Herrero R, Reyes RM et al. Risk factors for cancer of the oral cavity and oro-pharynx in Cuba. Br J Cancer 2001; 85: 46-54.

203. Ghani WMN, Razak IA, Doss JG et al. Multi-ethnic variations in the practice of oral cancer risk habits in a developing country. Oral Dis 2019; 25: 447-455.

204. Gholap D, Dikshit R, Chaturvedi P et al. Exclusive use of different types of tobacco products, exposure to secondhand tobacco smoke and risk of subtypes of head and neck cancer among Indian males. Int J Cancer 2023; 152: 374-383.

205. Gunasekera SK, Perera KA, Fernando C, Udagama PV. A shifting paradigm in the aetiology of oral and pharyngeal cancer in Sri Lanka: a case-control study providing serologic evidence for the role of oncogenic HPV types 16 and 18. Infect Agent Cancer 2015; 10: 12.

206. Horn-Ross PL, Ljung BM, Morrow M. Environmental factors and the risk of salivary gland cancer. Epidemiology 1997; 8: 414-419.

207. Hung HC, Chuang J, Chien YC et al. Genetic polymorphisms of CYP2E1, GSTM1, and GSTT1; environmental factors and risk of oral cancer. Cancer Epidemiol Biomarkers Prev 1997; 6: 901-905.

208. Ihsan R, Devi TR, Yadav DS et al. Investigation on the role of p53 codon 72 polymorphism and interactions with tobacco, betel quid, and alcohol in susceptibility to cancers in a high-risk population from North East India. DNA Cell Biol 2011; 30: 163-171.

209. Jussawalla DJ, Deshpande VA. Evaluation of cancer risk in tobacco chewers and smokers: an epidemiologic assessment. Cancer 1971; 28: 244-252.

210. Kabat GC, Wynder EL. Type of alcoholic beverage and oral cancer. Int J Cancer 1989; 43: 190-194.

211. Kabat GC, Hebert JR, Wynder EL. Risk factors for oral cancer in women. Cancer Res 1989; 49: 2803-2806.

212. Katoh T, Kaneko S, Kohshi K et al. Genetic polymorphisms of tobacco- and alcohol-related metabolizing enzymes and oral cavity cancer. Int J Cancer 1999; 83: 606-609.

213. Ko YC, Huang YL, Lee CH et al. Betel quid chewing, cigarette smoking and alcohol consumption related to oral cancer in Taiwan. J Oral Pathol Med 1995; 24: 450-453.

214. Kune GA, Kune S, Field B et al. Oral and pharyngeal cancer, diet, smoking, alcohol, and serum vitamin A and beta-carotene levels: a case-control study in men. Nutr Cancer 1993; 20: 61-70.

215. La Vecchia C, Bidoli E, Barra S et al. Type of cigarettes and cancers of the upper digestive and respiratory tract. Cancer Causes Control 1990; 1: 69-74.

216. Lakhanpal M, Yadav DS, Devi TR et al. Association of interleukin-1beta -511 C/T polymorphism with tobacco-associated cancer in northeast India: a study on oral and gastric cancer. Cancer Genet 2014; 207: 1-11.

217. Lee KW, Kuo WR, Tsai SM et al. Different impact from betel quid, alcohol and cigarette: risk factors for pharyngeal and laryngeal cancer. Int J Cancer 2005; 117: 831-836.

218. Lee CH, Lee KW, Fang FM et al. The neoplastic impact of tobacco-free betel-quid on the histological type and the anatomical site of aerodigestive tract cancers. Int J Cancer 2012; 131: E733-743.

219. Lee YA, Li S, Chen Y et al. Tobacco smoking, alcohol drinking, betel quid chewing, and the risk of head and neck cancer in an East Asian population. Head Neck 2019; 41: 92-102.

220. Lissowska J, Pilarska A, Pilarski P et al. Smoking, alcohol, diet, dentition and sexual practices in the epidemiology of oral cancer in Poland. Eur J Cancer Prev 2003; 12: 25-33.

221. Llewellyn CD, Johnson NW, Warnakulasuriya KA. Risk factors for oral cancer in newly diagnosed patients aged 45 years and younger: a case-control study in Southern England. J Oral Pathol Med 2004; 33: 525-532.

222. Llewellyn CD, Linklater K, Bell J et al. An analysis of risk factors for oral cancer in young people: a case-control study. Oral Oncol 2004; 40: 304-313.

223. Madani AH, Jahromi AS, Madhurina D, Denbanshu B. Risk as- sessment of tobacco types and oral cancer. AmJ Pharmacol Toxicol 2010; 5: 9-13.

224. Mahapatra S, Kamath R, Shetty BK, Binu VS. Risk of oral cancer associated with gutka and other tobacco products: a hospital-based case-control study. J Cancer Res Ther 2015; 11: 199-203.

225. Marques LA, Eluf-Neto J, Figueiredo RA et al. Oral health, hygiene practices and oral cancer. Rev Saude Publica 2008; 42: 471-479.

226. Marshall JR, Graham S, Haughey BP et al. Smoking, alcohol, dentition and diet in the epidemiology of oral cancer. Eur J Cancer B Oral Oncol 1992; 28B: 9-15.

227. Martinez I. Factors associated with ccer of the esophagus, mouth, and pharynx in Puerto Rico. J Natl Cancer Inst 1969; 42: 1069-1094.

228. Merletti F, Boffetta P, Ciccone G et al. Role of tobacco and alcoholic beverages in the etiology of cancer of the oral cavity/oropharynx in Torino, Italy. Cancer Res 1989; 49: 4919-4924.

229. Moreno-Lopez LA, Esparza-Gomez GC, Gonzalez-Navarro A et al. Risk of oral cancer associated with tobacco smoking, alcohol consumption and oral hygiene: a case-control study in Madrid, Spain. Oral Oncol 2000; 36: 170-174.

230. Maurya SS, Katiyar T, Dhawan A et al. Gene-environment interactions in determining differences in genetic susceptibility to cancer in subsites of the head and neck. Environ Mol Mutagen 2015; 56: 313-321.

231. Naghibzadeh-Tahami A, Karamoozian A, Iranpour A et al. Is opium use related to the increased risk of oral cavity cancers? A case-control study in Iran. Cancer Epidemiol 2024; 91: 102602.

232. Nandakumar A, Thimmasetty KT, Sreeramareddy NM et al. A population-based case-control investigation on cancers of the oral cavity in Bangalore, India. Br J Cancer 1990; 62: 847-851.

233. Oudjehih M, Deltour I, Bouhidel ML et al. Smokeless Tobacco Use, Cigarette Smoking, and Upper Aerodigestive Tract Cancers: A Case-Control Study in the Batna Region, Algeria, 2008-2011. Tob Use Insights 2020; 13: 1179173x20902239.

234. Radoï L, Menvielle G, Cyr D et al. Population attributable risks of oral cavity cancer to behavioral and medical risk factors in France: results of a large population-based case-control study, the ICARE study. BMC Cancer 2015; 15: 827.

235. Rodriguez T, Altieri A, Chatenoud L et al. Risk factors for oral and pharyngeal cancer in young adults. Oral Oncol 2004; 40: 207-213.

236. Sankaranarayanan R, Duffy SW, Day NE et al. A case-control investigation of cancer of the oral tongue and the floor of the mouth in southern India. Int J Cancer 1989; 44: 617-621.

237. Sapkota A, Gajalakshmi V, Jetly DH et al. Smokeless tobacco and increased risk of hypopharyngeal and laryngeal cancers: a multicentric case-control study from India. Int J Cancer 2007; 121: 1793-1798.

238. Schildt EB, Eriksson M, Hardell L, Magnuson A. Oral snuff, smoking habits and alcohol consumption in relation to oral cancer in a Swedish case-control study. Int J Cancer 1998; 77: 341-346.

239. Shewale JB, Pickard RKL, Xiao W et al. Independent association of marijuana use and poor oral hygiene with HPV-negative but not HPV-positive head and neck squamous cell carcinomas. Cancer 2021; 127: 2099-2110.

240. Smith EM, Hoffman HT, Summersgill KS et al. Human papillomavirus and risk of oral cancer. Laryngoscope 1998; 108: 1098-1103.

241. Smith EM, Rubenstein LM, Haugen TH et al. Tobacco and alcohol use increases the risk of both HPV-associated and HPV-independent head and neck cancers. Cancer Causes Control 2010; 21: 1369-1378.

242. Su WZ, Ohno Y, Tohnai I et al. Case-control study of oral cancer in Shenyang, Northeastern China. International Journal of Clinical Oncology 1998; 3: 13-18.

243. Subapriya R, Thangavelu A, Mathavan B et al. Assessment of risk factors for oral squamous cell carcinoma in Chidambaram, Southern India: a case-control study. Eur J Cancer Prev 2007; 16: 251-256.

244. Sugimura T, Kumimoto H, Tohnai I et al. Gene-environment interaction involved in oral carcinogenesis: molecular epidemiological study for metabolic and DNA repair gene polymorphisms. J Oral Pathol Med 2006; 35: 11-18.

245. Szymanska K, Hung RJ, Wunsch-Filho V et al. Alcohol and tobacco, and the risk of cancers of the upper aerodigestive tract in Latin America: a case-control study. Cancer Causes Control 2011; 22: 1037-1046.

246. Takezaki T, Hirose K, Inoue M et al. Tobacco, alcohol and dietary factors associated with the risk of oral cancer among Japanese. Jpn J Cancer Res 1996; 87: 555-562.

247. Takezaki T, Shinoda M, Hatooka S et al. Subsite-specific risk factors for hypopharyngeal and esophageal cancer (Japan). Cancer Causes Control 2000; 11: 597-608.

248. Tuyns AJ, Esteve J, Raymond L et al. Cancer of the larynx/hypopharynx, tobacco and alcohol: IARC international case-control study in Turin and Varese (Italy), Zaragoza and Navarra (Spain), Geneva (Switzerland) and Calvados (France). Int J Cancer 1988; 41: 483-491.

249. Varela-Lema L, Ruano-Ravina A, Juiz Crespo MA, Barros-Dios JM. Tobacco consumption and oral and pharyngeal cancer in a Spanish male population. Cancer Lett 2010; 288: 28-35.

250. Vlajinac HD, Marinkovic JM, Sipetic SB et al. Case-control study of oropharyngeal cancer. Cancer Detect Prev 2006; 30: 152-157.

251. Wang D, Ritchie JM, Smith EM et al. Alcohol dehydrogenase 3 and risk of squamous cell carcinomas of the head and neck. Cancer Epidemiol Biomarkers Prev 2005; 14: 626-632.

252. Wasnik KS, Ughade SN, Zodpey SP, Ingole DL. Tobacco consumption practices and risk of oro-pharyngeal cancer: a case-control study in Central India. Southeast Asian J Trop Med Public Health 1998; 29: 827-834.

253. Werbrouck J, De Ruyck K, Duprez F et al. Single-nucleotide polymorphisms in DNA double-strand break repair genes: association with head and neck cancer and interaction with tobacco use and alcohol consumption. Mutat Res 2008; 656: 74-81.

254. Winn DM, Blot WJ, Shy CM et al. Snuff dipping and oral cancer among women in the southern United States. N Engl J Med 1981; 304: 745-749.

255. Yang Z, Sun P, Dahlstrom KR et al. Joint effect of human papillomavirus exposure, smoking and alcohol on risk of oral squamous cell carcinoma. BMC Cancer 2023; 23: 457.

256. Zavras AI, Douglass CW, Joshipura K et al. Smoking and alcohol in the etiology of oral cancer: gender-specific risk profiles in the south of Greece. Oral Oncol 2001; 37: 28-35.

257. Zeng J, Tang Y, Wu P et al. Alcohol consumption, tobacco smoking, betel quid chewing and oral health associations with hypopharyngeal cancer among men in Central South China: a case-control study. Cancer Manag Res 2019; 11: 6353-6364.

258. Znaor A, Brennan P, Gajalakshmi V et al. Independent and combined effects of tobacco smoking, chewing and alcohol drinking on the risk of oral, pharyngeal and esophageal cancers in Indian men. Int J Cancer 2003; 105: 681-686.

259. Agudo A, Bonet C, Travier N et al. Impact of cigarette smoking on cancer risk in the European prospective investigation into cancer and nutrition study. J Clin Oncol 2012; 30: 4550-4557.

260. Akiba S, Hirayama T. Cigarette smoking and cancer mortality risk in Japanese men and women--results from reanalysis of the six-prefecture cohort study data. Environ Health Perspect 1990; 87: 19-26.

261. Akiba S. Analysis of cancer risk related to longitudinal information on smoking habits. Environ Health Perspect 1994; 102 Suppl 8: 15-19.

262. Blakely T, Barendregt JJ, Foster RH et al. The association of active smoking with multiple cancers: national census-cancer registry cohorts with quantitative bias analysis. Cancer Causes Control 2013; 24: 1243-1255.

263. Christensen CH, Rostron B, Cosgrove C et al. Association of Cigarette, Cigar, and Pipe Use With Mortality Risk in the US Population. JAMA Intern Med 2018; 178: 469-476.

264. Freedman ND, Abnet CC, Leitzmann MF et al. Prospective investigation of the cigarette smoking-head and neck cancer association by sex. Cancer 2007; 110: 1593-1601.

265. Freedman ND, Abnet CC, Caporaso NE et al. Impact of changing US cigarette smoking patterns on incident cancer: risks of 20 smoking-related cancers among the women and men of the NIH-AARP cohort. Int J Epidemiol 2016; 45: 846-856.

266. Friborg JT, Yuan JM, Wang R et al. A prospective study of tobacco and alcohol use as risk factors for pharyngeal carcinomas in Singapore Chinese. Cancer 2007; 109: 1183-1191.

267. Hsu WL, Chien YC, Chiang CJ et al. Lifetime risk of distinct upper aerodigestive tract cancers and consumption of alcohol, betel and cigarette. Int J Cancer 2014; 135: 1480-1486.

268. Ide R, Mizoue T, Fujino Y et al. Cigarette smoking, alcohol drinking, and oral and pharyngeal cancer mortality in Japan. Oral Dis 2008; 14: 314-319.

269. Jacob L, Freyn M, Kalder M et al. Impact of tobacco smoking on the risk of developing 25 different cancers in the UK: a retrospective study of 422,010 patients followed for up to 30 years. Oncotarget 2018; 9: 17420-17429.

270. Liaw KM, Chen CJ. Mortality attributable to cigarette smoking in Taiwan: a 12-year follow-up study. Tob Control 1998; 7: 141-148.

271. Lu Y, Sobue T, Kitamura T et al. Cigarette smoking, alcohol drinking, and oral cavity and pharyngeal cancer in the Japanese: a population-based cohort study in Japan. Eur J Cancer Prev 2018; 27: 171-179.

272. Maasland DH, van den Brandt PA, Kremer B et al. Alcohol consumption, cigarette smoking and the risk of subtypes of head-neck cancer: results from the Netherlands Cohort Study. BMC Cancer 2014; 14: 187.

273. McLaughlin JK, Hrubec Z, Blot WJ, Fraumeni JF, Jr. Smoking and cancer mortality among U.S. veterans: a 26-year follow-up. Int J Cancer 1995; 60: 190-193.

274. Mezzoiuso AG, Odone A, Signorelli C, Russo AG. Association Between Smoking And Cancers Among Women: Results From The FRiCaM Multisite Cohort Study. J Cancer 2021; 12: 3136-3144.

275. Morales DR, Pacurariu A, Slattery J, Kurz X. Association between hydrochlorothiazide exposure and different incident skin, lip and oral cavity cancers: A series of population-based nested case-control studies. Br J Clin Pharmacol 2020; 86: 1336-1345.

276. Muwonge R, Ramadas K, Sankila R et al. Role of tobacco smoking, chewing and alcohol drinking in the risk of oral cancer in Trivandrum, India: a nested case-control design using incident cancer cases. Oral Oncol 2008; 44: 446-454.

277. Nordlund LA, Carstensen JM, Pershagen G. Cancer incidence in female smokers: a 26-year follow-up. Int J Cancer 1997; 73: 625-628.

278. Offermans NS, Vermeulen R, Burdorf A et al. Occupational asbestos exposure and risk of oral cavity and pharyngeal cancer in the prospective Netherlands Cohort Study. Scand J Work Environ Health 2014; 40: 420-427.

279. Pednekar MS, Gupta PC, Yeole BB, Hébert JR. Association of tobacco habits, including bidi smoking, with overall and site-specific cancer incidence: results from the Mumbai cohort study. Cancer Causes Control 2011; 22: 859-868.

280. Saito N, Sairenchi T, Irie F et al. Duration of cigarette smoking is a risk factor for oropharyngeal cancer mortality among Japanese men and women: the Ibaraki Prefectural Health Study (IPHS). Ann Epidemiol 2013; 23: 546-550.

281. Wen CP, Tsai SP, Chen CJ, Cheng TY. The mortality risks of smokers in Taiwan: Part I: cause-specific mortality. Prev Med 2004; 39: 528-535.

282. Yun YH, Jung KW, Bae JM et al. Cigarette smoking and cancer incidence risk in adult men: National Health Insurance Corporation Study. Cancer Detect Prev 2005; 29: 15-24.
