# Supplementary material for: Smoking and oral and pharyngeal cancer: a meta-analysis
Source: Oncol Rev. 2026 Jan 20;19:1672607. doi: 10.3389/or.2025.1672607 (PMC12865294; doi:10.3389/or.2025.1672607)
Supplement: Supplementary file 2 [file Supplementaryfile2.docx]

**Smoking and Oral and Pharyngeal Cancer: A Meta-Analysis**

**Annex – 1**

***Search strategy***

As a first step, we conducted an umbrella review focusing on the association between smoking and SHS exposure and cancer risk in different anatomical sites. We performed a literature search of PubMed/MEDLINE, Embase, the Institute for Scientific Information Web of Science, and the Cochrane Database of Systematic Reviews, in order to identify relevant meta-analyses, pooled analyses, and systematic reviews on the association between cigarette smoking and SHS exposure and cancer risk up to 12 October 2022.

The umbrella review identified 61 reports on the association between cigarette smoking and the risk of cancers located anywhere in the upper aerodigestive tract **(Supplementary Table 1)**, including systematic reviews, meta-analyses, pooled analyses, or reports from international agencies. All original articles addressing OPC were extracted from these reports, resulting in the identification of 195 non-duplicate original publications on tobacco smoking and the risk of OPC. We included cancers of the oral cavity (ICD-10: C00-C09), oropharynx (ICD-10: C10), and hypopharynx (ICD-10: C13). Given the complex aetiology of nasopharyngeal cancer (ICD-10: C11), which distinguishes it from other pharyngeal cancers, considering it within the analysis of overall pharyngeal sites is not appropriate. Therefore, articles that focused only on nasopharyngeal cancer were excluded and analysed in a separate meta-analysis ^1^. Selected articles on OPC were screened based on their full text using the eligibility criteria described in the following section, resulting in the exclusion of 94 articles identified as ineligible **(Supplementary Table 2).**

In the following phase of our search, we conducted a traditional literature search in PubMed/MEDLINE and Embase that included all original studies published between January 2008 (i.e., the beginning of the year of the search string of the most recent and comprehensive review available on the topic ^2^) and 5 February 2025. Our search string included combinations of MeSH terms and text words related to OPC and tobacco or smoking **(Supplementary Box 1)**. After excluding duplicate publications and ineligible articles, the literature update resulted in 43 original publications on cigarette smoking and the risk of OPC. Six articles retrieved during the conduction of previous systematic reviews on other cancer sites were also added.

By merging the original articles identified in the umbrella review and the traditional review, a total of 137 non-duplicate publications were considered eligible (**Supplementary Figure 1)**.

***Eligibility criteria***

To be included in this meta-analysis, studies had to meet specific eligibility criteria: i) be either case-control studies (including nested case-control studies or pooled analyses of case-control studies) or cohort studies (including case-cohort studies or pooled analyses of cohort studies); ii) be published as original articles in the English language; iii) provide data on the general population; iv) provide information on the association between cigarette smoking and the risk of oral cancer, pharyngeal (excluding nasopharyngeal) cancer, or both combined; (v) report risk estimates, including risk ratios, odds ratios, hazard ratios, or mortality rate ratios - all referred to as relative risk (RR) - for at least one variable among smoking status (current, former, and/or ever smokers), intensity, duration, and time since quitting, compared with never or current cigarette smokers, and the corresponding 95% confidence intervals (CI), or provide sufficient information to calculate them.

***Data extraction***

Information, including both general and study-specific details, was systematically collected from each eligible study during the data extraction phase. Data extracted included: general publication information (e.g., first author, year of publication, and journal), study characteristics (e.g., country in which the study was conducted, study name, study design, and sample size), details of the statistical model used for RR estimates (including covariates considered), and RRs with corresponding 95% Cis. Where available, data on the number of cases and persons at risk/person-years (for cohort studies) or the number of controls (for case-control studies) were extracted for each smoking exposure category (e.g., never, current, former, or ever smokers). Where available, numbers of cases and controls (or persons at risk/person-years for cohort studies) for each smoking exposure category (e.g., never, current, former, or ever smokers) and for dose-response analyses (e.g., categories of smoking intensity, duration, or time since quitting).

Where appropriate, we used the technique described by Hamling and colleagues for aggregating non-independent estimates ^3^. This approach involved modification of the reference category or collapsing of RRs from two or more categories in studies where the reference group was the same across categories. Where RRs were reported separately for different anatomical subsites of OPC, we used the method described by Rucker and colleagues ^4^ to obtain a single RR for overall OPC.

***Statistical analysis***

To account for heterogeneity of risk estimates, we used random effects meta-analytic models ^5^.

Heterogeneity was assessed using the χ^2^ test, and inconsistency was quantified using the I^2^ statistic, which represents the proportion of total variation attributable to between-study variance ^6^. We carried out stratified analyses based on various study and population characteristics, such as cancer anatomical and histological subtype, sex, study design, type of control (for case-control studies), endpoint (for cohort studies), tertiles of the number of cases, presence of adequate adjustments, study quality, geographic area, income group, and year of publication.

Study quality was assessed using the Newcastle-Ottawa Scale (NOS) ^7^. The NOS score ranges from 0 (poor quality) to 9 (good quality) and considers information on three broad categories: selection (maximum 4 points), comparability (maximum 2 points), and outcome for case-control or exposure for cohort studies (maximum 3 points). In this meta-analysis, high-quality studies were defined as those with NOS scores ≥7. To ensure the completeness and comprehensiveness of our study, no low-quality study was excluded from the meta-analysis.

To investigate publication bias, we examined funnel plots ^8^ and employed Egger’s test for funnel plot asymmetry ^9^. Publication bias was further explored using the Duval and Tweedie trim-and-fill method to estimate adjusted pooled effect sizes ^10^.

We examined both linear and nonlinear associations between smoking intensity (for current vs. never smokers), smoking duration (for current vs. never smokers), and time since quitting (for former vs. current smokers) and the log RR of OPC. Dose-response relationships were evaluated using a one-stage random-effects dose-response model ^11^. Nonlinear coefficients were subjected to the Wald test for statistical significance. When linearity was rejected, nonlinear relationships were modelled using restricted cubic splines with three knots at fixed percentiles of exposure (10%, 50%, and 90%) ^12,13^. Exposure levels for each category were determined as the midpoint between upper and lower bounds; for open-ended upper categories, exposure levels were set at 1.2 times the lower bound ^14,15^.

When the number of cases and/or controls for a specific exposure category was not available in the original published studies, we estimated the covariance between log RR by considering the total number of cases and/or controls in the studies, weighted by the average percentage distribution of subjects pooled from all other studies ^16^.

**References**

1. Possenti I, Martini A, Bagnardi V, et al. Association between cigarette smoking and nasopharyngeal cancer risk: a meta-analysis. *Rhinology.* 2025;63(1):13-21.

2. IARC. Personal habits and indoor combustions. Volume 100 E. *IARC Monogr Eval Carcinog Risks Hum.* 2012:1-538.

3. Hamling J, Lee P, Weitkunat R, Ambuhl M. Facilitating meta-analyses by deriving relative effect and precision estimates for alternative comparisons from a set of estimates presented by exposure level or disease category. *Stat Med.* 2008;27(7):954-970.

4. Rucker G, Cates CJ, Schwarzer G. Methods for including information from multi-arm trials in pairwise meta-analysis. *Res Synth Methods.* 2017;8(4):392-403.

5. DerSimonian R, Laird N. Meta-analysis in clinical trials. *Control Clin Trials.* 1986;7(3):177-188.

6. Higgins JP, Thompson SG. Quantifying heterogeneity in a meta-analysis. *Stat Med.* 2002;21(11):1539-1558.

7. Wells GA, Shea B, O'Connell D, et al. The Newcastle-Ottawa Scale (NOS) for assessing the quality of nonrandomised studies in meta-analyses (accessed 23 November 2023). 2014.

8. Peters JL, Sutton AJ, Jones DR, Abrams KR, Rushton L. Contour-enhanced meta-analysis funnel plots help distinguish publication bias from other causes of asymmetry. *J Clin Epidemiol.* 2008;61(10):991-996.

9. Egger M, Davey Smith G, Schneider M, Minder C. Bias in meta-analysis detected by a simple, graphical test. *BMJ.* 1997;315(7109):629-634.

10. Duval S, Tweedie R. Trim and fill: A simple funnel-plot-based method of testing and adjusting for publication bias in meta-analysis. *Biometrics.* 2000;56(2):455-463.

11. Crippa A, Discacciati A, Bottai M, Spiegelman D, Orsini N. One-stage dose-response meta-analysis for aggregated data. *Stat Methods Med Res.* 2019;28(5):1579-1596.

12. Desquilbet L, Mariotti F. Dose-response analyses using restricted cubic spline functions in public health research. *Stat Med.* 2010;29(9):1037-1057.

13. Lugo A, Peveri G, Bosetti C, et al. Strong excess risk of pancreatic cancer for low frequency and duration of cigarette smoking: A comprehensive review and meta-analysis. *Eur J Cancer.* 2018;104:117-126.

14. Bagnardi V, Rota M, Botteri E, et al. Alcohol consumption and site-specific cancer risk: a comprehensive dose-response meta-analysis. *Br J Cancer.* 2015;112(3):580-593.

15. Berlin JA, Longnecker MP, Greenland S. Meta-analysis of epidemiologic dose-response data. *Epidemiology.* 1993;4(3):218-228.

16. Crippa A, Orsini A.,. Multivariate Dose-Response Meta-Analysis: The dosresmeta R Package. 2016.
